# Global hypermethylation of intestinal epithelial cells is a hallmark feature of neonatal surgical necrotizing enterocolitis

**DOI:** 10.1186/s13148-020-00983-6

**Published:** 2020-12-11

**Authors:** Misty Good, Tianjiao Chu, Patricia Shaw, Lora McClain, Austin Chamberlain, Carlos Castro, Jamie M. Rimer, Belgacem Mihi, Qingqing Gong, Lila S. Nolan, Krista Cooksey, Laura Linneman, Pranjal Agrawal, David N. Finegold, David Peters

**Affiliations:** 1 Department of Pediatrics, Division of Newborn Medicine, Washington University School of Medicine/St. Louis Children’s Hospital, 660 S. Euclid Ave. Campus, Box 8208, St. Louis, MO 63110 USA; 2 Departments of Obstetrics, Gynecology and Reproductive Sciences, University of Pittsburgh, 204 Craft Avenue, Pittsburgh, PA 15213 USA; 3 Human Genetics, University of Pittsburgh, Pittsburgh, PA USA; 4 Psychiatry, University of Pittsburgh, Pittsburgh, PA USA; 5 Magee-Womens Research Institute, Pittsburgh, PA USA; 6 Present Address: PathGroup, Brentwood, TN USA

**Keywords:** DNA methylation, Epigenetics, Necrotizing enterocolitis, Intestinal epithelium, Neonatal

## Abstract

**Background:**

Necrotizing enterocolitis (NEC) remains one of the overall leading causes of death in premature infants, and the pathogenesis is unpredictable and not well characterized. The aim of our study was to determine the molecular phenotype of NEC via transcriptomic and epithelial cell-specific epigenomic analysis, with a specific focus on DNA methylation.

**Methods:**

Using laser capture microdissection, epithelial cell-specific methylation signatures were characterized by whole-genome bisulfite sequencing of ileal and colonic samples at the time of surgery for NEC and after NEC had healed at reanastomosis (*n* = 40). RNA sequencing was also performed to determine the transcriptomic profile of these samples, and a comparison was made to the methylome data.

**Results:**

We found that surgical NEC has a considerable impact on the epigenome by broadly increasing DNA methylation levels, although these effects are less pronounced in genomic regions associated with the regulation of gene expression. Furthermore, NEC-related DNA methylation signatures were influenced by tissue of origin, with significant differences being noted between colon and ileum. We also identified numerous transcriptional changes in NEC and clear associations between gene expression and DNA methylation.

**Conclusions:**

We have defined the intestinal epigenomic and transcriptomic signatures during surgical NEC, which will advance our understanding of disease pathogenesis and may enable the development of novel precision medicine approaches for NEC prediction, diagnosis and phenotyping.
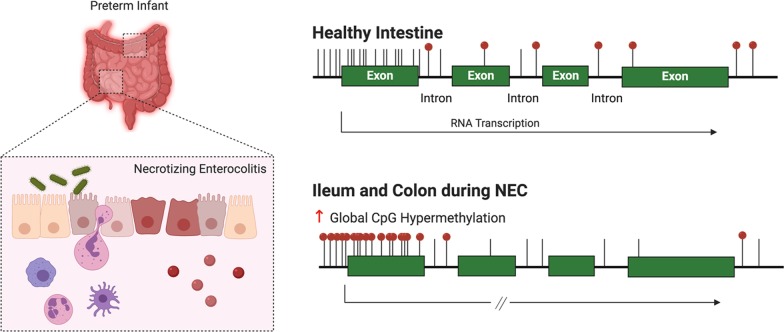

## Introduction

Necrotizing enterocolitis remains a leading cause of death in premature infants [1–3] for which there remain limited treatment options, no reliable biomarkers and the pathogenesis remains unpredictable. NEC can rapidly take its toll on a growing premature infant to an urgent need of cardiorespiratory support leading to intestinal resection for necrotic bowel a few hours later. The precise disease mechanisms leading to surgical NEC (sNEC) remain ill defined, likely in part because few intestinal samples are available for analysis. In seeking to advance the understanding of NEC pathogenesis, several authors have performed whole genome microarray analysis [4] or bulk RNA sequencing on intestinal resections from infants with NEC [5, 6]. While these studies have offered some additional insights into the gene expression profiles of infants with surgical NEC, there is considerable variability in the samples. To combat this limitation, we developed the NEC biorepository [7, 8], which provides us with the unique opportunity to advance the field of NEC research with a larger number of samples for sequencing and analysis. These intestinal samples will help to further identify the precise molecular signaling pathways involved in the pathogenesis of sNEC, with the hope to identify infants at the highest risk for the development of this devastating disease.

High-throughput genomic sequencing approaches have enormous potential with regard to unraveling complex heterogeneous phenotypes at the molecular level. One approach is to evaluate the DNA methylation of cytosine–guanine dinucleotides (CpGs) in the promoter, exons, introns, enhancers and CpG island shore regions of genes as they can have a significant impact on the regulation of gene expression. Alterations in the epigenomic molecular phenotype at the level of DNA methylation have been frequently associated with profound transcriptional changes leading to disease pathobiology [9, 10]. As we continue toward the era of precision medicine, we sought to apply this approach to infants in the neonatal intensive care unit (NICU) with NEC. Since epithelial cell signaling during NEC has been an important factor in deciphering the pathogenesis of the disease [11, 12], we focused this study on the epithelial cell methylome signature. Here we determined the epigenomic molecular phenotype of surgical NEC via genome-wide DNA methylation analysis. Specifically, we identified DNA methylation differences in intestinal epithelial cell genomic DNA within resected ileum or colon that may provide new insights into the biology and mechanisms of NEC pathogenesis.

## Results

**Fig. 1:**
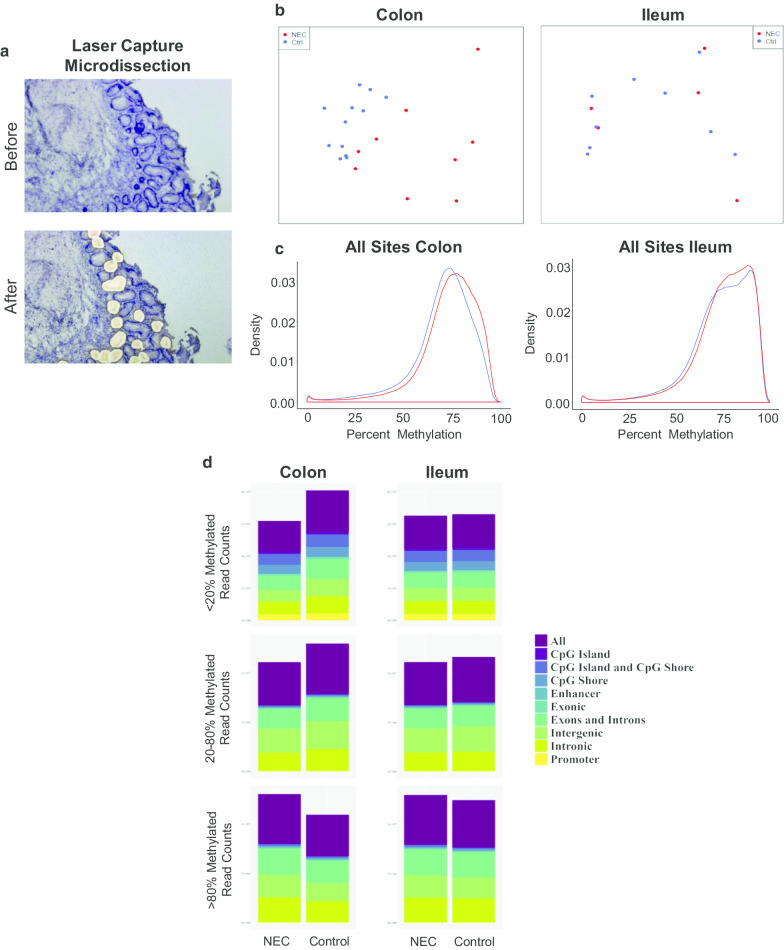
**a** Representative images of neonatal gut epithelial cells before (left) and after (right) laser capture microdissection (LCM). **b** Classic multidimensional scaling of WGBS data from NEC and non-NEC colon (left) and ileum (right). **c** Density plots of CpG methylation levels across the entire genome using WGBS data from NEC or non-NEC colon (left) or ileum (right) (see also Additional file 2: Figure S1). Red lines represent NEC colon/ileum, and blue lines represent non-NEC colon/ileum. **d** Distribution of differentially methylated NEC-specific CpG sites falling into (top) low methylation (LM) (< 20%), (middle) intermediate methylation (IM) (20–80%) and (lower) high methylation (HM) (> 80%) categories across all sites and different genomic elements and combinations thereof

We prepared a total of *n* = 40 bisulfite sequencing libraries from individual tissue samples following laser capture microdissection of intestinal epithelial cells in each case. These consisted of *n* = 12 from non-NEC colon samples, *n* = 10 from sNEC colon, *n* = 11 from non-NEC ileum samples and *n* = 7 from sNEC ileum. Representative images of neonatal gut epithelial cells before and after LCM are shown as an example in Fig. 1a. The resulting DNA samples were subjected to whole-genome bisulfite sequencing (WGBS) in which we sequenced a total of 5,162,557,910 aligned read pairs across all samples. Summary statistics for WGBS sequencing data are presented in Additional file 1: Table S1A.

### Hypermethylation of gut epithelium is observed in sNEC

We first sought to identify broad differences in sNEC versus non-NEC samples in both the large intestine (colon) and small intestine (ileum). Multidimensional scaling of these data revealed a clear separation between sNEC and non-NEC samples in colon, but less pronounced global differences between sNEC ileum and non-NEC ileum (Fig. 1b). To further examine the broad patterns of CpG methylation, we generated density plots of CpG methylation levels across the entire genome. These report the global distribution of CpG methylation levels without reference to genomic location. Density plots revealed a global shift toward higher levels of methylation in sNEC colon compared to non-NEC colon (Additional file 2: Figure S1A). This relationship was observed across CpG sites in promoters, exons, introns, intergenic regions, CpG island shores and enhancers, but was less clear in CpG islands (CGIs). We also identified similar, albeit less pronounced, global differences when sNEC ileum was compared to non-NEC ileal samples with the exception that CGI shores and promoters showed little or no difference in the overall methylation level between sNEC and non-NEC ileal samples (Additional file 2: Figure S1B).

On further analysis, we identified CpG sites falling into low methylation (LM) (< 20%), intermediate methylation (IM) (20–80%) and high methylation (HM) (> 80%) categories and found that for both non-NEC ileum and non-NEC colon, approximately 2% of sites were categorized as LM with the remainder being distributed relatively evenly between IM and HM sites. These distributions were similar for both ileal and colonic sNEC samples, although in both cases, we found that proportions of LM and IM sites were reduced and HM sites were increased in NEC (*p* ≤ 0.0001) (Fig. 1c, Tables 1, 2). Further analysis of distinct genomic elements revealed that these distributions are also seen in exons and introns and similarly altered between sNEC and non-NEC samples. In contrast, LM sites account for far greater proportions of CGIs, promoters, CGI shores and, to a lesser extent, enhancers in both colon and ileum. Although the distributions of these were altered in both sample types between sNEC and non-NEC samples for promoters and CGI shores (*p* ≤ 0.0001), no such differences were observed between sNEC and non-NEC CGIs (Fig. 1c, Tables 1, 2).

**Table 1:** Distribution of low, intermediate and highly methylated DNA methylation sites between different genomic elements in NEC and non-NEC ileum tissues

Feature	Methylation	Ileum				
	Percent	NEC (%)^a^	Non-NEC (%)^a^	Chi-square^2^^b^	*df*	*p*
Promoter	< 20	37,570 (21.3)	37,809 (21.4)	5.1	2	0.079
	20–80	67,544 (38.2)	67,963 (38.5)			
	> 80	71,628 (40.5)	70,970 (40.2)			
Exonic	< 20	11,252 (4.7)	11,276 (4.7)	83.9	2	< 0.0001
	20–80	105,227 (43.6)	108,315 (44.9)			
	> 80	124,986 (51.8)	121,874 (50.5)			
Intronic	< 20	83,095 (1.9)	84,504 (1.9)	2270	2	< 0.0001
	20–80	1,880,459 (42.3)	1,949,492 (43.8)			
	> 80	2,483,522 (55.8)	2,413,080 (54.3)			
CpG Island	< 20	14,248 (45.5)	14,297 (45.6)	0.32	2	0.85
	20–80	6478 (20.7)	6496 (20.7)			
	> 80	10,611 (33.9)	10,544 (33.6)			
CpG Shore	< 20	55,456 (15.9)	55,885 (16.0)	9.49	2	0.0087
	20–80	125,323 (35.9)	124,102 (35.6)			
	> 80	168,110 (48.2)	168,902 (48.4)			
Enhancer	< 20	895 (7.6)	910 (7.7)	2.55	2	0.28
	20–80	5807 (49.4)	5913 (50.3)			
	> 80	5047 (43.0)	4926 (41.9)			
Intergenic	< 20	80,112 (1.7)	82,289 (1.7)	7668	2	< 0.0001
	20–80	2,462,179 (51.2)	2,595,021 (54.0)			
	> 80	2,264,520 (47.1)	2,129,501 (44.3)			
CpG Island + CpG Shore	< 20	69,704 (18.3)	70,182 (18.5)	8.62	2	0.014
	20–80	131,801 (34.7)	130,598 (34.3)			
	> 80	178,721 (47.0)	179,446 (47.2)			
Exons + Introns	< 20	94,347 (2.0)	95,780 (2.0)	2349	2	< 0.0001
	20–80	1,985,686 (42.4)	2,057,807 (43.9)			
	> 80	2,608,508 (55.6)	2,534,954 (54.1)			
All	< 20	205,779 (2.1)	208,548 (2.2)	12,306	2	< 0.0001
	20–80	4,378,720 (45.3)	4,618,095 (47.8)			
	> 80	5,074,456 (52.5)	4,832,312 (50.0)			

*df* degrees of freedom, *p* probability

^a^Count (percent)

^b^Pearson's Chi-squared test

**Table 2:** Distribution of low, intermediate and highly methylated DNA methylation sites between different genomic elements in NEC and non-NEC colon tissues

Feature	Methylation	Colon				
	Percent	NEC (%)^a^	Non-NEC (%)^a^	Chi-square^2^^b^	*df*	*p*
Promoter	< 20	37,158 (21.0)	41,311 (23.4)	937	2	< 0.0001
	20–80	66,839 (37.8)	71,457 (40.4)			
	> 80	72,745 (41.2)	63,974 (36.2)			
Exonic	< 20	11,149 (4.6)	12,921 (5.4)	3926	2	< 0.0001
	20–80	103,599 (42.9)	123,568 (51.2)			
	> 80	126,717 (52.5)	104,976 (43.5)			
Intronic	< 20	78,867 (1.8)	110,129 (2.5)	65,796	2	< 0.0001
	20–80	1,849,085 (41.6)	2,196,549 (49.4)			
	> 80	2,519,124 (56.6)	2,140,398 (48.1)			
CpG Island	< 20	14,441 (46.1)	14,521 (46.3)	1.1	2	0.57
	20–80	6607 (21.1)	6651 (21.2)			
	> 80	10,289 (32.8)	10,165 (32.4)			
CpG Shore	< 20	54,460 (15.6)	62,519 (17.9)	1362	2	< 0.0001
	20–80	123,299 (35.3)	129,751 (37.2)			
	> 80	171,130 (49)	156,619 (44.9)			
Enhancer	< 20	913 (7.8)	1095 (9.3)	89	2	< 0.0001
	20–80	5830 (49.6)	6339 (54.0)			
	> 80	5006 (42.6)	4315 (36.7)			
Intergenic	< 20	72,468 (1.5)	106,195 (2.2)	74,182	2	< 0.0001
	20–80	2,472,995 (51.4)	2,850,249 (59.3)			
	> 80	2,261,348 (47.0)	1,850,367 (38.5)			
CpG Island + CpG Shore	< 20	68,901 (18.1)	77,040 (20.3)	1228	2	< 0.0001
	20–80	129,906 (34.2)	136,402 (35.9)			
	> 80	181,419 (47.7)	166,784 (43.9)			
Exons + Introns	< 20	90,016 (1.9)	123,050 (2.6)	69,507	2	< 0.0001
	20–80	1,952,684 (41.6)	2,320,117 (49.5)			
	> 80	2,645,841 (56.4)	2,245,374 (47.9)			
All	< 20	191,444 (2.0)	261,637 (2.7)	145,070	2	< 0.0001
	20–80	4,407,199 (45.6)	5,166,249 (53.5)			
	> 80	5,060,312 (52.4)	4,231,069 (43.8)			

*df* degrees of freedom, *p* probability

^a^Count (percent)

^b^Pearson's Chi-squared test

**Fig. 2:**
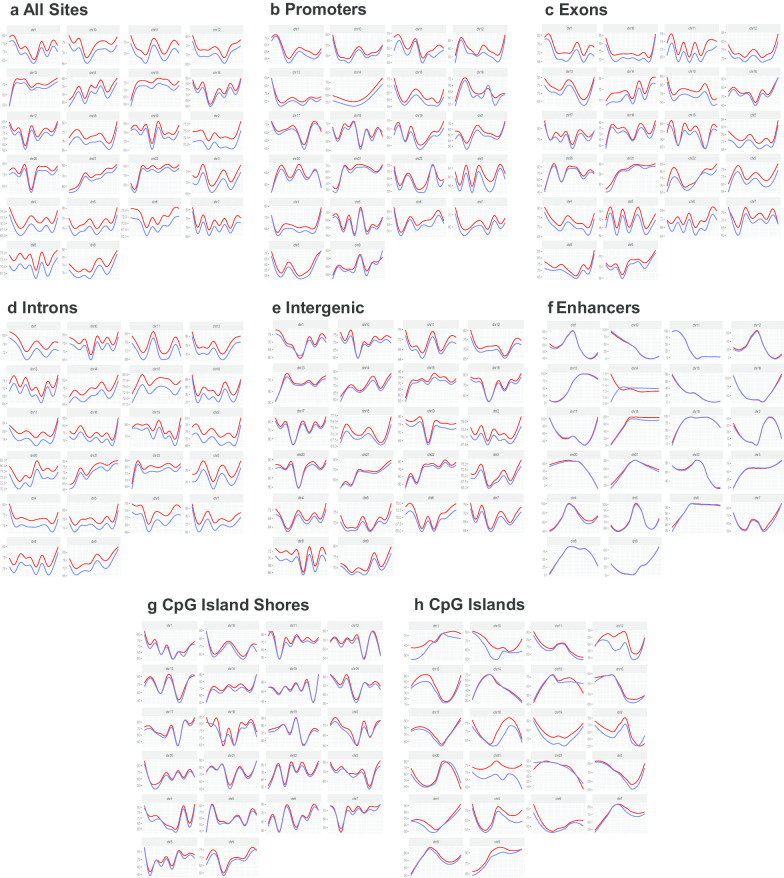
CpG methylation levels mapped spatially across all autosomes. Red lines represent NEC colon, and blue lines represent non-NEC colon. Data are presented for **a** all sites, **b** promoters, **c** exons, **d** introns, **e** intergenic regions, **f** enhancers, **g** CpG island shores and **h** CpG islands (CGI)

We next mapped CpG methylation across each autosome, which allows CpG methylation patterns to be visualized spatially. We plotted methylation levels across all human autosomes for each group of samples (sNEC and non-NEC colon and ileum) (Fig. 2, Additional file 2: Figure S2). These analyses confirmed the relative global hypermethylation of sNEC colon when compared to non-NEC colon. We also confirmed relative hypermethylation of sNEC colon when considering CpG sites in promoters, exons, introns, intergenic regions, enhancers, CGIs and CGI shores (Fig. 2). Similar, though less pronounced, trends were noted when sNEC ileal samples were compared with non-NEC ileal samples (Additional file 2: Figure S2).

### Identification of NEC-specific differentially methylated regions: pathway analysis and tissue specificity

**Fig. 3:**
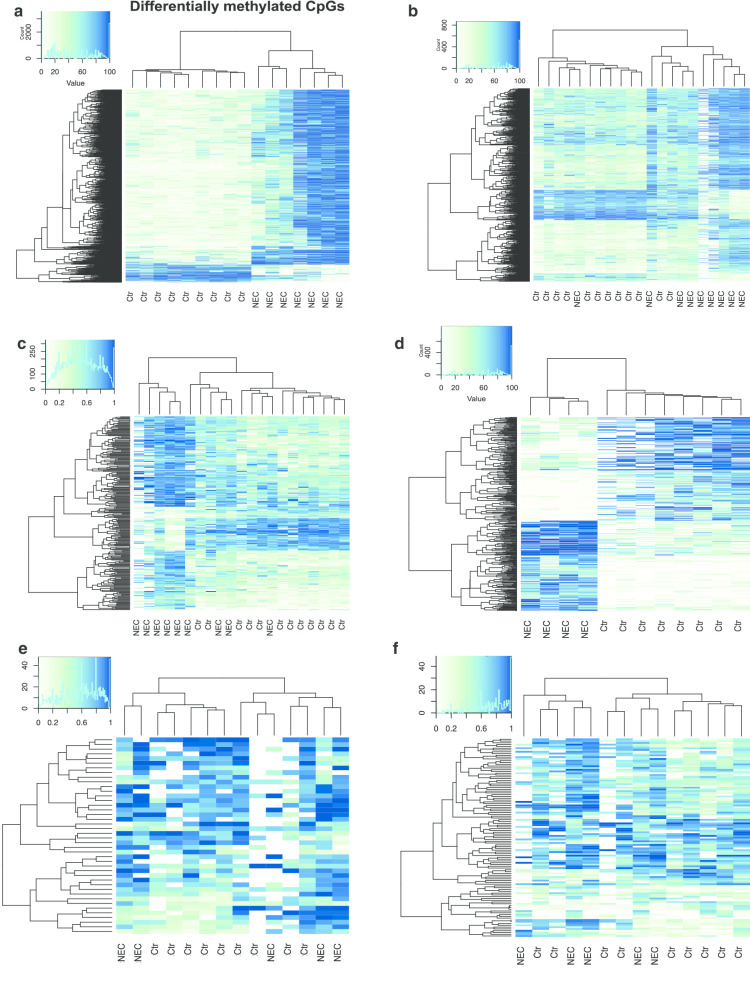
Differentially methylated CpG regions (DMRs) that differ between NEC and non-NEC colon and NEC and non-NEC ileum. **a** Autosomal DMRs with a difference in average methylation rate of at least 0.1 between NEC and non-NEC (Ctr) colon samples. **b** NEC-specific colon DMRs in promoter regions and **c** gene bodies. **d** Autosomal DMRs with a difference in average methylation rate of at least 0.1 between NEC and non-NEC ileum samples. **e** NEC-specific ileum DMRs in promoter regions and **f** gene bodies

Our analysis identified differentially methylated CpG regions (DMRs) that differ between sNEC and non-NEC colon and ileum. Specifically, we identified 5265 autosomal DMRs with a difference in average methylation rate of at least 0.1 (10%) between sNEC and non-NEC colon samples (Fig. 3a). These included 4212 in promoter regions (Fig. 3b, Additional file 1: Table S2A) and 1627 DMRs in gene bodies (introns/exons) (Fig. 3c, Additional file 1: Table S2B). Furthermore, we identified 2785 autosomal DMRs in sNEC versus non-NEC ileum samples with a difference in average methylation rate of at least 0.1 (Fig. 3d). These included 2223 promoter in regions (Fig. 3e, Additional file 1: Table S2C) and 840 in gene bodies (Fig. 3f, Additional file 1: Table S2D). Further analysis revealed that 197 DMRs were shared between colon and ileum (Additional file 1: Table S2E).

Previous studies have demonstrated that DNA methylation signatures can reveal considerable information regarding the molecular phenotype of the tissue/cell type(s) in question [13–15]. We therefore explored the functions of genes in which sNEC-specific DMRs were identified using Ingenuity Pathways Analysis (IPA) software. We performed IPA analysis on genes whose average promoter methylation rates are altered in sNEC colon samples versus non-NEC samples. We hypothesized that this approach might identify small effects that are broadly shared between multiple CpG sites within promoters. Promoter regions in which average CpG methylation is significantly altered between sNEC and non-NEC colon samples (Additional file 1: Table S2B) were used to identify enriched pathways as described above. These include “role of pattern recognition receptors in the recognition of bacteria and viruses” (*p* = 8.44 × 10^–4^), “leukocyte extravasation signaling” (*p* = 1.28 × 10^–3^), “triacylglycerol biosynthesis” (*p* = 2.33 × 10^–3^), role of hypercytokinemia/hyperchemokinemia in the pathogenesis of influenza (*p* = 1.28 × 10^–3^) and “interferon signaling” (*p* = 3.71 × 10^–3^) (Additional file 2: Figure S3, Figure S4A-D and Additional file 1: Table S3A). We also found that the most highly significant predicted upstream regulators of the identified promoters were hepatocyte nuclear factor 4 alpha (HNF4A) (1.20 × 10^–9^) and HNF1A (3.42 × 10^–4^). Putative HNF4A/HNFA1 target genes within our data, as identified by IPA analysis, are shown in Additional file 1: Tables S3C and D and Additional file 2: Figures S5A and B, respectively. Similar analyses were performed for sNEC-specific promoter DMRs identified in the ileum. These analyses identified a number of significant pathways, including “granulocyte adhesion and diapedesis” (1.34 × 10^–2^), “induction of apoptosis by HIV1” (1.35 × 10^–2^), “IL-17 signaling” (1.69 × 10^–2^) and “CD40 signaling” (1.81 × 10^–2^) (Additional file 2: Figure S6A-D and Additional file 1: Table S3B).

### Identification of sNEC-specific differentially methylated CpG sites: pathway analysis and tissue specificity

We identified differentially methylated single CpG sites (DMSs) and found that there were 38,809 of these between laser-captured epithelium from sNEC and non-NEC colon with an adjusted *p* value (*q* value) of < 0.05 (*p* value < 0.00043) (Additional file 1: Table S4A). Notably, there were far fewer of these (*n* = 652) at the same level of significance (*q* = 0.05) when comparing sNEC and non-NEC ileum (Additional file 1: Table S4B). The 30 DMSs displaying the most highly significant differences (*q* value) between sNEC and non-NEC colon and sNEC and non-NEC ileum are shown in Tables 3 and 4, respectively.

**Table 3:** Top 30 differentially methylated single CpG sites (DMSs) between NEC and non-NEC colon

chr	Start	End	*p* value	*q* value	meth.diff	feature.name	name2	dist.to.feature	feature.strand	Prom	Exon	Intron	mydiff.CpGi.annot.members	mydiff.shores.annot.members	mydiff.enhancer.annot.members
chr17	11019015	11019015	1.20E−21	8.39 E−15	40.29664	NM_001101387	PIRT	− 180,915	–	0	0	0	0	0	0
chr2	86062716	86062716	2.67 E−17	9.34 E−11	32.23282	NM_015425	POLR1A	43,440	–	0	0	1	0	0	0
chr4	115491856	115491856	5.48 E−16	1.28 E−09	40.79545	NM_022569	NDST4	− 377,981	–	0	0	0	0	0	0
chr14	52272903	52272903	1.95 E−15	3.41 E−09	72.84615	NM_001281469	PTGDR	5192	+	0	0	1	0	0	0
chr10	21066470	21066470	1.40 E−14	1.32 E−08	35.83795	NM_001177483	C10orf113	80,090	–	0	0	1	0	0	0
chr3	175224788	175224788	1.33 E−14	1.32 E−08	37.83784	NR_046713	NAALADL2-AS2	46,309	–	0	0	1	0	0	0
chr5	24263140	24263140	1.51 E−14	1.32 E−08	56.26401	NR_131245	C5orf17	311,794	+	0	0	0	0	0	0
chr4	58967547	58967547	2.02 E−14	1.57 E−08	38.96104	NR_133941	LINC02429	− 16,735	+	0	0	0	0	0	0
chr2	79966760	79966760	3.05 E−14	2.14 E−08	55.40583	NR_107047	MIR8080	− 100,178	–	0	0	1	0	0	0
chr4	22556420	22556420	3.43 E−14	2.18 E−08	45.32618	NM_145290	ADGRA3	− 40,367	–	0	0	0	0	0	0
chr5	17911285	17911285	3.99 E−14	2.33 E−08	33.63636	NR_134287	LINC02223	104,013	+	0	0	1	0	0	0
chr2	119409733	119409733	4.60 E−14	2.47 E−08	53.94235	NM_183240	TMEM37	− 22,137	+	0	0	0	0	0	0
chr20	38460167	38460167	9.79 E−14	4.89 E−08	42.20401	NR_002986	SNORA60	10,800	+	0	0	0	0	0	0
chr3	101804213	101804213	1.31 E−13	5.91 E−08	51.44188	NM_001005474	NFKBIZ	− 23,777	+	0	0	1	0	0	0
chr7	67694175	67694175	1.35 E−13	5.91 E−08	64.74501	NR_120514	LOC102723427	− 326,078	+	0	0	0	0	0	0
chr6	121426895	121426895	1.44 E−13	5.94 E−08	63.50051	NM_000165	GJA1	− 8682	+	0	0	0	0	0	0
chr12	101416276	101416276	1.81 E−13	7.02 E−08	42.5265	NM_001301068	ARL1	− 8457	–	0	0	0	0	0	0
chr6	78594584	78594584	2.13 E−13	7.85 E−08	50.29663	NM_001010844	IRAK1BP1	− 272,960	+	0	0	0	0	0	0
chr11	12359400	12359400	2.47 E−13	8.05 E−08	38.54167	NM_018222	PARVA	− 18,171	+	0	1	0	0	0	0
chr15	63678136	63678136	2.37 E−13	8.05 E−08	55.5917	NR_034080	USP3-AS1	− 77,310	–	0	1	0	0	0	0
chr6	81046717	81046717	2.53 E−13	8.05 E−08	31.96195	NM_017633	TENT5A	705,995	–	0	0	0	0	0	0
chr1	167097455	167097455	3.50 E−13	1.02 E−07	66.47619	NM_001080426	DUSP27	3367	+	0	0	1	0	0	0
chr15	81380112	81380112	4.30 E−13	1.20 E−07	44.66705	NM_001080532	TMC3	− 5957	–	0	0	1	0	0	0
chr2	150152356	150152356	4.63 E−13	1.25 E−07	56.97802	NR_146972	LINC01817	104,652	–	0	0	0	0	0	0
chr6	167440526	167440526	6.28 E−13	1.47 E−07	47.95134	NR_134591	LOC105378127	32,265	–	0	0	0	0	0	0
chr16	63305874	63305874	7.40 E−13	1.67 E−07	40.34462	NM_001796	CDH8	− 1E+06	–	0	0	0	0	0	0
chr10	99591101	99591101	8.02 E−13	1.70 E−07	60.53068	NM_031212	SLC25A28	29,364	–	0	0	0	0	0	0
chr5	22585518	22585518	7.92 E−13	1.70 E−07	50.73758	NM_001364104	CDH12	267,827	–	0	0	1	0	0	0
chr8	61783188	61783188	8.72 E−13	1.80 E−07	58.48694	NR_039680	MIR4470	68,402	+	0	0	0	0	0	0
chr19	53612831	53612831	1.15 E−12	2.24 E−07	51.38783	NR_002938	LOC284379	− 9335	–	0	0	0	0	0	0

**Table 4:** Top 30 differentially methylated single CpG sites (DMSs) between NEC and non-NEC ileum

chr	Start	End	*p* value	*q*value	meth.diff	feature.name	name2	dist.to.feature	feature.strand	Prom	Exon	Intron	mydiff.CpGi.annot.members	mydiff.shores.annot.members	mydiff.enhancer.annot.members
chr2	70827641	70827641	2.11E−23	1.85 E−16	− 95.2406	NM_001258027	CLEC4F	− 7042	−	0	0	0	0	0	0
chr1	100785163	100785163	4.60 E−21	2.02 E−14	− 52.5346	NM_080682	VCAM1	65,525	+	0	0	0	0	0	0
chr1	100762620	100762620	2.21 E−17	6.47 E−11	− 59.1667	NM_080682	VCAM1	42,982	+	0	0	0	0	0	0
chr1	143641173	143641173	6.27 E−17	1.38 E−10	34.04255	NR_104078	RNVU1-17	58,447	–	0	0	0	0	1	0
chr6	73558525	73558525	9.22 E−17	1.62 E−10	84.61538	NM_001402	EEF1A1	− 37,494	–	0	0	0	0	0	0
chr11	10964709	10964709	5.72 E−16	8.36 E−10	39.43662	NR_034137	ZBED5-AS1	106,494	+	0	0	0	0	0	0
chr17	53706550	53706550	4.09 E−15	5.13 E−09	− 71.4286	NM_032559	KIF2B	− 116,328	+	0	0	0	0	0	0
chr1	100785162	100785162	1.64 E−14	1.80 E−08	− 47.6821	NM_080682	VCAM1	65,524	+	0	0	0	0	0	0
chr10	53230713	53230713	2.29 E−13	2.23 E−07	36	NM_000242	MBL2	− 459,014	–	0	0	0	0	0	0
chr8	27077249	27077249	3.38 E−13	2.96 E−07	94.11765	NR_031680	MIR548H4	− 28,287	–	0	0	0	0	0	0
chr5	36566389	36566389	5.11 E−13	4.08 E−07	31.16883	NM_001289940	SLC1A3	− 39,966	+	0	0	0	0	0	0
chr5	60868950	60868950	6.00 E−13	4.18 E−07	− 72.093	NM_001104558	ELOVL7	− 24,677	–	0	0	0	0	0	0
chr7	29003503	29003503	6.20 E−13	4.18 E−07	35.71429	NR_038965	LOC100506497	23,538	+	0	0	1	0	0	0
chr1	100768520	100768520	1.11 E−12	6.93 E−07	− 55.7692	NM_080682	VCAM1	48,882	+	0	0	0	0	0	0
chr11	47812794	47812794	1.21 E−12	7.05 E−07	− 86.4865	NM_015231	NUP160	35,751	–	0	0	1	0	0	0
chr4	147431181	147431181	1.47 E−12	8.06 E−07	− 95.2381	NR_045958	EDNRA	− 49,736	+	0	0	0	0	0	0
chr1	67009294	67009294	1.81 E−12	9.33 E−07	− 57.8231	NM_015139	SLC35D1	45,104	–	0	0	1	0	0	0
chr2	45853090	45853090	2.60 E−12	1.27 E−06	94.44444	NM_005400	PRKCE	201,188	+	0	0	1	0	0	0
chr13	33070399	33070399	3.26 E−12	1.51 E−06	− 70.7317	NM_004795	KL	53,968	+	0	0	0	0	0	0
chr14	68071519	68071519	4.29 E−12	1.88 E−06	− 80.0909	NM_001321815	RAD51B	247,929	+	0	0	1	0	0	0
chr13	25087158	25087158	4.95 E−12	2.07 E−06	− 88.8889	NM_030979	PABPC3	− 8980	+	0	0	0	0	0	0
chr1	100750518	100750518	9.64 E−12	3.68 E−06	− 58.5586	NM_080682	VCAM1	30,880	+	0	0	0	0	0	0
chr14	22548745	22548745	9.48 E−12	3.68 E−06	73.91304	NR_146543	LINC02332	8318	–	0	0	0	0	0	0
chr19	7737210	7737210	1.05 E−11	3.84 E−06	− 41.0526	NM_198492	CLEC4G	− 5040	–	0	0	0	0	0	0
chr2	54973902	54973902	1.29 E−11	4.52 E−06	30.64516	NM_007008	RTN4	36,433	–	0	0	1	0	0	0
chr5	61059457	61059457	1.71 E−11	5.78 E−06	− 59.7015	NR_109908	SMIM15-AS1	− 102,859	+	0	0	1	0	0	0
chr1	123215253	123215253	1.80 E−11	5.86 E−06	35.55556	NR_003955	EMBP1	1,696,143	+	0	0	0	0	0	0
chr5	116099451	116099451	1.91 E−11	5.86 E−06	− 88.3333	NM_001308080	COMMD10	13,991	+	0	0	1	0	0	0
chr6	33559051	33559051	1.94 E−11	5.86 E−06	− 76.087	NM_001188	BAK1	21,243	–	0	0	0	0	0	0
chr3	78587902	78587902	2.03 E−11	5.94 E−06	− 85.7143	NM_133631	ROBO1	431,558	–	0	0	0	0	0	0

We validated a subset of DMSs via multiplex PCR and next-generation sequencing of bisulfite converted DNA. As shown in Additional file 2: Figure S5C, we found high correlation between primary WGBS data and the targeted follow-up analyses. Genomic coordinates and methylation levels for the sites assayed are shown in Additional file 1: Table S5.

We performed IPA analysis of DMSs within gene promoters whose DNA methylation levels were significantly altered between laser-captured epithelium from sNEC and non-NEC colon (*q* ≤ 0.05). Ileum data were not included in these analyses due to the relatively small number of differences identified at *q* = 0.05. We identified statistically significant enrichment of gene promoters in various pathways, upstream regulators and known disease phenotypes. For example, we found that the promoters of genes containing CpG sites whose methylation levels were significantly altered in sNEC versus control colon samples were enriched in pathways involving “integrin signaling” (*p* = 2.32 × 10^–10^), “molecular mechanisms of cancer” (*p* = 1.62 × 10^–8^), “ERK/MAPK signaling” (*p* = 1.21 × 10^–6^), “leukocyte extravasation signaling” (*p* = 2.05 × 10^–6^) and “caveolar-mediated endocytosis signaling” (*p* = 2.06 × 10^–6^) (Additional file 2: Figure S7, Figure S8A-D and Additional file 1: Table S6). As before, hepatocyte nuclear factor 4 alpha (HNF4A) (*p* = 2.11 × 10^–7^) was identified as a likely upstream regulator of these gene promoters. Given that DNA methylation in CGI shores has been shown to be associated with transcription, we also explored functional associations of genes containing differentially expressed DMSs in CGI shores. We found that these were enriched in a number of pathways including “molecular mechanisms of cancer” (*p* = 3.07 × 10^–7^), “AMPK signaling” (*p* = 3.01 × 10^–5^), PDGF signaling (*p* = 9.58 × 10^–5^), “PPARα/RXRα activation” (*p* = 1.6 × 10–4) and “p53 signaling” (*p* = 3.99 × 10–4) (Additional file 2: Figure S9, Figure S10A-D and Additional file 1: Table S7A).

Given the clear pattern of sNEC-associated hypermethylation in our data, we explored the methylation levels of a number of key DNA methyltransferases and associated factors. Notably, we found that CpG sites located within the upstream region of DNMT3A were broadly hypermethylated in sNEC compared to non-NEC controls. Similar observations were made upstream of TET2 and within an intron of TET3. We also found that CpG sites within intronic regions of DNMT3B and DNMT3L were significantly less methylated in sNEC than in non-NEC control (Additional file 1: Table S7B).

### Analysis of NEC-specific transcriptional changes in neonatal intestine

**Fig. 4:**
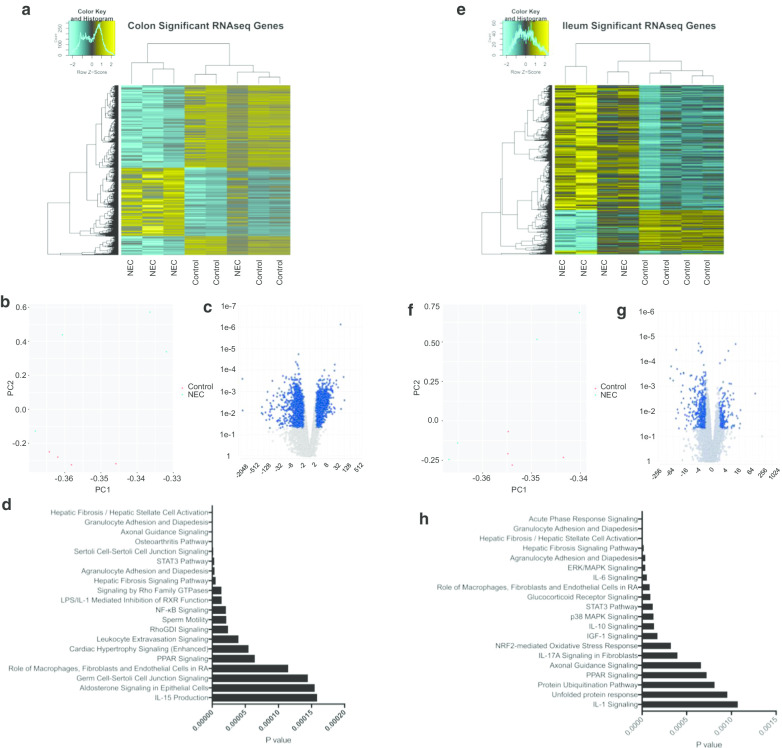
RNA sequencing of mRNA from **a**–**d** colon and **e**–**h** ileum of NEC and control subjects. **a**, **e** Heat map of significant (*p* ≤ 0.05, least squares [LS] mean > 1) differences in RNA expression between NEC and non-NEC **a** colon or **e** ileum. **b**, **f** Principle component analysis of RNA expression in NEC and non-NEC **b** colon and **f** ileum. **c**, **g** Volcano plot of **c** colon or **g** ileum RNA expression fold change (X-axis) and *p* value (Y-axis). Highlighted dots represent genes that have a *p* value of less than 0.05 and a fold change of at least 2. **d**, **h** The 20 most significant results from functional pathway analysis of differentially expressed genes in **d** colon or **h** ileum

**Fig. 5:**
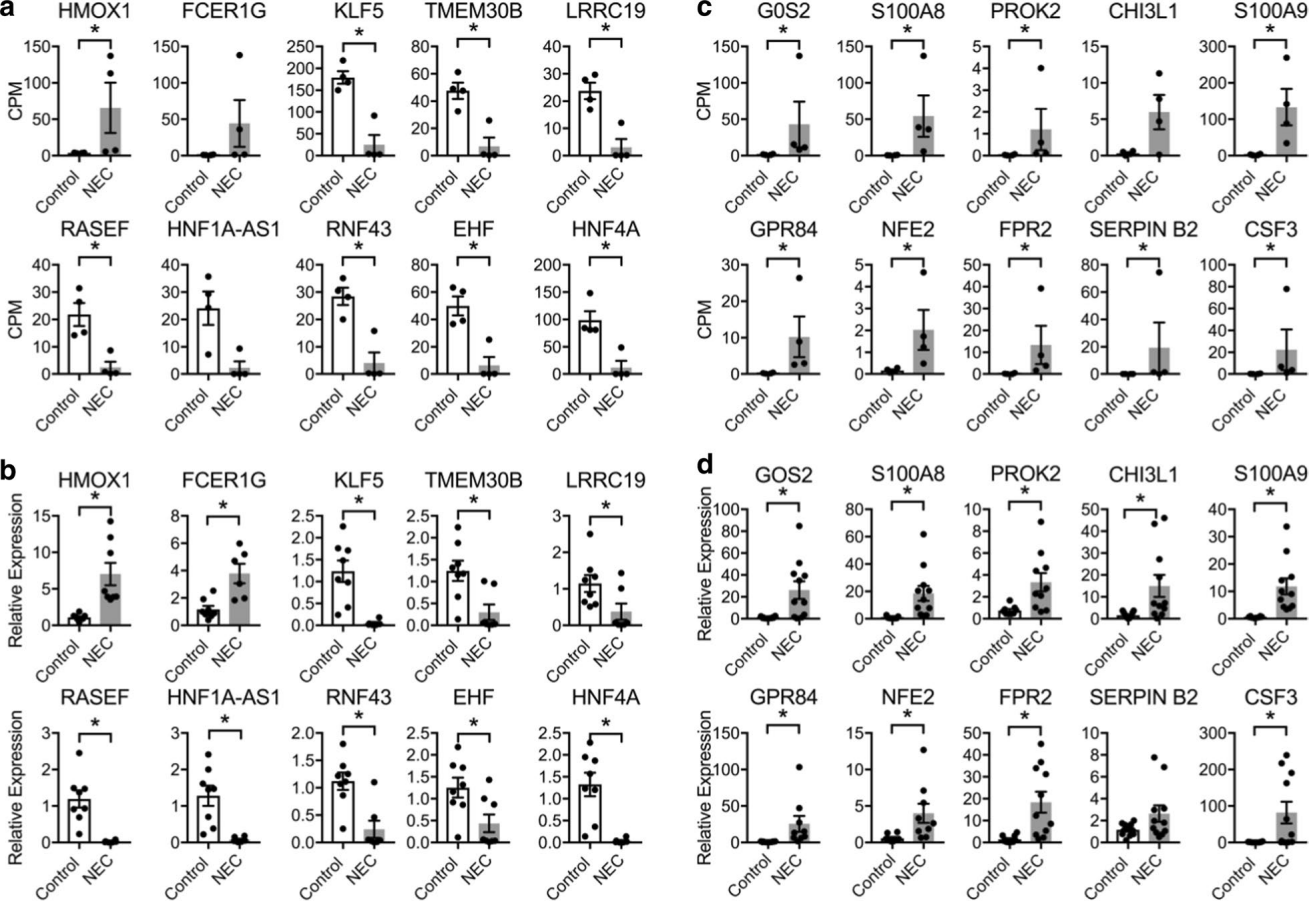
Changes in mRNA levels of selected genes in **a**, **b** colon and **c**, **d** ileum of control and NEC subjects. **a**, **c** RNA-seq (*n* = 4) read counts normalized to counts per millions of reads in each sample. **b**, **d** qPCR (*n* = 11) data are presented as relative expression normalized to RPLP0. *p < 0.05

We used RNA sequencing to evaluate the transcriptional signature of human colon (Fig. 4a–d) and ileum (Fig. 4e–h) during active sNEC compared to non-NEC controls. Using a significance cutoff of *p* ≤ 0.05, least squares (LS) mean > 1, we identified 1760 mRNAs (37.3%) whose expressions were elevated in sNEC colon versus non-NEC control colon samples and 2596 mRNAs (62.7%) whose expressions were reduced (Fig. 4a, Additional file 1: Table S8A). In contrast, we identified 649 mRNAs (75.7%) whose expressions were elevated in sNEC ileum versus non-NEC control ileum samples and 208 mRNAs (24.3%) whose expressions were reduced (Fig. 4e, Additional file 1: Table S8B). sNEC-specific mRNA expression changes were confirmed for a number of transcripts by quantitative RT-PCR (Fig. 5a–d).

Functional pathway analysis of differentially expressed transcripts identified in the colon revealed enrichment of genes in pathways for “hepatic fibrosis/hepatic stellate activation” (*p* = 3.68 × 10^–10^), “granulocyte adhesion and diapedesis” (*p* = 8.76 × 10^–9^) and “axonal guidance” (*p* = 4.98 × 10^–8^). These results are summarized in Additional file 2: Figure S11A-C, Figure S12A-C and Additional file 1: Table S9. Functional pathway analysis of differentially expressed transcripts in ileum revealed enrichment of genes in pathways for “acute phase response signaling” (*p* = 3.84 × 10^–8^), “granulocyte adhesion and diapedesis” (*p* = 4.24 × 10^–8^) and “hepatic fibrosis/hepatic stellate activation” (*p* = 1.46 × 10^–6^). These results are summarized in Additional file 2: Figure S11D-F, Figure S13A-C and Additional file 1: Table S10. It is notable that the enriched pathway, “granulocyte adhesion and diapedesis,” was also found to be significantly enriched in genes containing ileal promoter DMRs (see above). Importantly, in the setting of inflammation, leukocytes can migrate through the blood vessel endothelium to the site of intestinal injury.

### DNA methylation changes in sNEC are associated with altered RNA expression patterns

We next searched for overlap between the protein coding RNAs that are differentially expressed (*p* ≤ 0.05) in sNEC versus non-NEC colon and the corresponding DMRs present in our sNEC versus non-NEC colon data. Similar comparisons were made for the ileal samples. We found 739 such mRNAs encoded by genes containing sNEC DMRs in colon (Additional file 1: Table S11A). Fewer such mRNAs were identified in sNEC ileum (*n* = 71) (Additional file 1: Table S11B). Functional analysis of colon data using IPA revealed enrichment in a number of pathways including endothelin 1 signaling (*p* = 2.70 × 10^–4^), corticotropin releasing hormone signaling (*p* = 1.37 × 10^–3^) and hepatic cholestasis (2.62 × 10^–3^) (Additional file 2: Figure S14, Additional file 1: Table S12). Importantly, HNF4 was again identified as a potential upstream regulator of the genes identified in colon and the HNF4 promoter was found to be hypermethylated in NEC colon, while the expression of its corresponding RNA was reduced (eightfold reduction in sNEC colon relative to non-NEC). Notably, HNF4A was identified as being a likely upstream regulator of genes containing sNEC-specific differentially methylated CpGs (see above). Other potential upstream regulators of genes whose expressions and DNA methylation levels are altered in sNEC colon include HNF1A/B and TNF (Additional file 2: Figure S14, Additional file 1: Table S13).

In the majority of colon-specific genes, in which differential expression was correlated with differential methylation, expression was reduced in sNEC colon compared to non-NEC colon (505/739 [68.3%]). With respect to these down-modulated genes, we found that their corresponding promoter regions were more frequently hypermethylated in NEC colon tissues compared to non-NEC controls (428/505 [84.8%]). In contrast, hypermethylation was less frequent in genes with elevated expression in sNEC colon (153/234 [65.4%]). Thus, sNEC colon-specific decreases in gene expression were associated with hypermethylation in corresponding promoters and, concomitantly, promoter hypomethylation was associated with increased gene expression in sNEC.

These findings were confirmed when we compared colon sNEC-specific mRNA expression data to colon sNEC-specific DMS data. We found 7087 differentially methylated CpG sites (*q* ≤ 0.05) that were within or adjacent to genes encoding mRNAs whose expressions were altered (*p* ≤ 0.05, LS mean > 1) between sNEC and non-NEC colon (Additional file 1: Table S14). Of these, approximately 63% DMSs (4474/7087) were found in genes whose expressions were reduced in sNEC colon compared to non-NEC controls. Furthermore, 92% (183/198) of DMSs located within promoters of genes whose expressions were decreased in NEC colon were found to display sNEC-specific hypermethylation, whereas only 66% (63/95) of those located in promoters of genes with increased expression in sNEC colon displayed hypermethylation. A similar trend was observed with respect to CGI shores with ~ 92% (428/467) of DMSs in CGIs found to be hypermethylated in genes whose expressions were decreased in sNEC colon compared to ~ 73% (120/164) within genes whose expressions were increased. As with DMRs, these DMS-specific results suggest that hypermethylation of promoter and CGI shores may influence the regulation of sNEC-specific RNA expression.

**Fig. 6:**
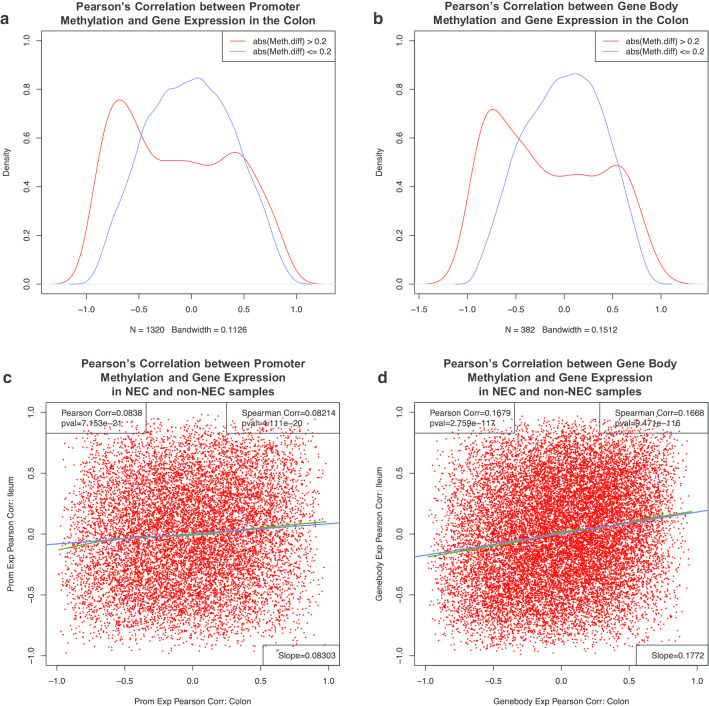
Density plots of Pearson’s correlation coefficients between **a** promoter (**b**) gene body methylation and gene expression in colon. **c** Scatter plot of Pearson’s correlation between promoter methylation and gene expression in NEC and non-NEC colon samples (X-axis) and Pearson’s correlation between promoter methylation and gene expression in NEC and non-NEC ileum samples (Y-axis). **d** Scatter plot of Pearson’s correlation coefficients between gene body methylation and gene expression in NEC and non-NEC colon samples (X-axis) and Pearson’s correlation between gene body methylation and gene expression in NEC and non-NEC ileum samples (Y-axis)

To further explore these relationships between DNA methylation and gene expression in sNEC, we plotted the distribution of the Pearson’s correlation between promoter or gene body methylation and gene expression in colon samples. In Fig. 6, the red lines represent genes for which the methylation difference between sNEC and non-NEC colon samples > 0.2 (20%), and the blue represents genes for which the difference ≤ 0.2. Clearly for genes with large differences in promoter and gene body methylation between sNEC and non-NEC colon, there was a significant negative correlation between gene expression and promoter (Fig. 6a) and gene body (Fig. 6b) methylation. This suggests that sNEC-specific expression changes are likely to be caused (in part) by changes in promoter and gene body methylation levels in some genes.

We next plotted Pearson’s correlation between promoter methylation and gene expression in sNEC and non-NEC colon samples (X-axis in Fig. 6c) and Pearson’s correlation between promoter methylation and gene expression in sNEC and non-NEC ileum samples (Y-axis in Fig. 6c). This identified a small, but statistically very significant correlation between the two sets of correlations, suggesting that when gene expression and promoter methylation are positively correlated in colon samples, there is a slightly higher chance that the gene expression and promoter methylation of that gene are also positively correlated in ileum samples. Furthermore, when gene expression and promoter methylation were negatively correlated in colon samples, we found it was more likely that the gene expression and promoter methylation of that gene were also negatively correlated in ileum samples. Similarly, we also plotted Pearson’s correlation between gene body methylation and gene expression in sNEC and non-NEC colon samples (X-axis in Fig. 6d) and Pearson’s correlation between gene body methylation and gene expression in sNEC and non-NEC ileum samples (Y-axis in Fig. 6d). We found a clear correlation between the two sets of analyses, suggesting that when gene expression and gene body methylation are positively correlated in colon samples, there is a higher chance that the gene expression and gene body methylation of that gene will also be positively correlated in ileum samples.

**Fig. 7:**
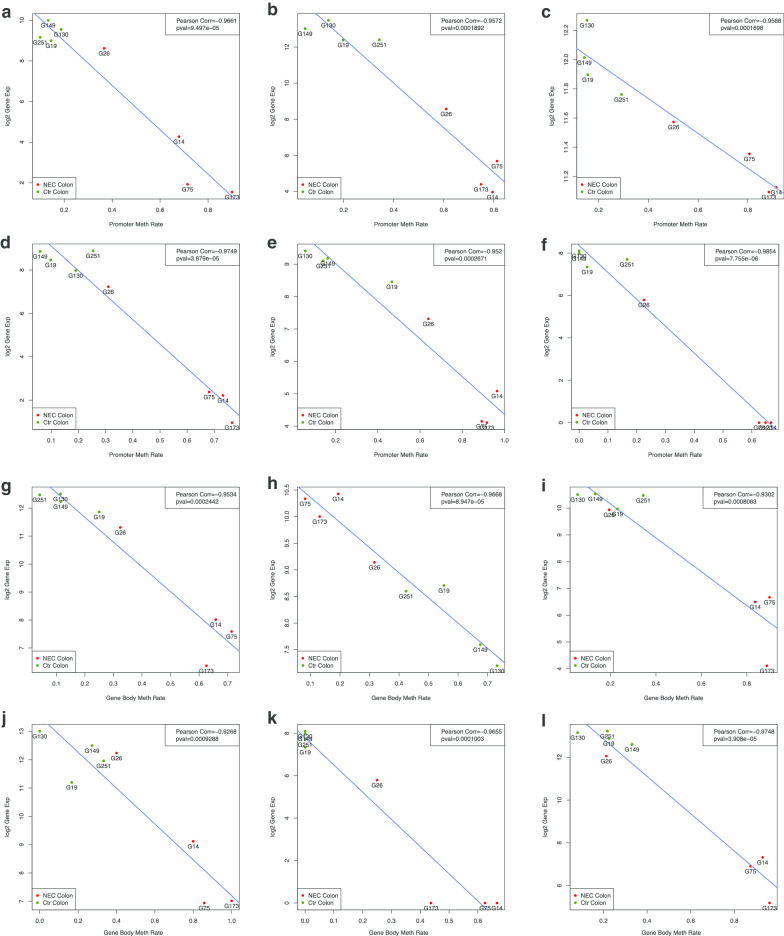
Identification of genes whose RNA expressions are most closely associated with DNA methylation in promoters (**a**–**f**) and gene bodies (**g**–**l**). **a** TINAG, **b** NXPE4, **c** PAK1, **d** CNGA1, **e** SAMD13, **f** LINC02038, **g** ESRP2, **h** HOXB2, **i** REP15, **j** GPX2, **k** LINC02038 and **l** MIR194-2HG. We plotted promoter methylation rate against log2 normalized gene expression. The genes were selected based on the following criteria: (1) their *p* values of the empirical Bayes *t* test (using R package limma) of logit-transformed promoter methylation between NEC and control colons ≤ 0.05; (2) the difference in logit-transformed promoter methylation between NEC and control colons ≥ 1; (3), the adjusted *p* values of the *t* test for Pearson’s correlation between gene expression and promoter methylation ≤ 0.1; and (4) those with adjusted *p* values ≤ 0.05

Finally, to identify genes whose expressions are most closely associated with DNA methylation, we plotted promoter methylation rate against log2 normalized gene expression. We selected a number of genes meeting the following criteria: (1) their *p* values of the empirical Bayes *t *test (using R package limma) of logit-transformed promoter methylation between sNEC and control colons ≤ 0.05; (2) the difference in logit-transformed promoter methylation between sNEC and control colons ≥ 1; (3), the adjusted *p* values of the t test for Pearson’s correlation between gene expression and promoter methylation ≤ 0.1; and (4) those with adjusted *p* values ≤ 0.05. Selected genes are shown in Fig. 7. Of the genes we identified, some have already been described as being functionally important in complex intestinal disease phenotypes. For example, the expression of TINAG is highly restricted to the kidney, colon, duodenum and small intestine. Neurexophilin and PC-Esterase Domain Family, Member 4 (NXPE4), which has biased expression in colon, is potentially associated with ulcerative colitis [16], as is P21 activated protein kinase 1 (PAK1) [17, 18]. Taken together, these findings reveal that the intestinal epithelial cell methylome and the transcriptome are significantly altered in the pathogenesis of sNEC.

## Discussion

We present a comprehensive analysis of the impact of sNEC on the epigenetic signatures of neonatal colonic and ileal epithelial cells. In addition to providing global insight into the structure and organization of the DNA methylome in the context of promoters, CGIs, CGI shores, enhancers, introns, exons and promoters, we present novel findings regarding the biological pathways that may be impacted by sNEC-associated epigenetic changes and the association between sNEC-specific DNA methylation and sNEC-specific RNA expression signatures. Our data are significant because whole genome bisulfite sequencing was performed in an epithelial cell-specific manner after laser capture microdissection of neonatal sNEC and non-NEC colon and ileal epithelium. Thus, the data are cell type-specific and minimally influenced by dilution effects that can be caused by the analysis of genome equivalents from complex mixtures of multiple cell types that may be present in differing proportions during bulk RNA sequencing. Such cell type-specific analyses are particularly important because DNA methylation is influenced significantly by cell lineage [19, 20].

Our data show that sNEC-derived epithelial cells are broadly hypermethylated in a genome-wide fashion relative to their non-NEC counterparts. This trend was evident when data were analyzed via density plots to examine overall levels of CpG methylation, when data were analyzed spatially and when methylation levels were separated by CpG site into low, intermediate and high categories. The hypermethylation of sNEC versus non-NEC colon was clearly evident when considering CpG sites present in each of introns, exons, intergenic regions, promoters and, to some extent, enhancers. Interestingly, however, it was not evident when considering only CpGs located within CGIs and CGI shores, which displayed no obvious difference.

Not surprisingly, we identified a multitude of sNEC-specific differentially methylated regions (DMRs) and differentially methylated sites (DMSs) in both colon and ileum. Further prospective investigations are needed to determine the timing of onset of the methylation changes seen during NEC and whether these modifications are detectable in the stool prior to NEC onset. These future studies will also examine the association of infant sex and age on DNA methylation signatures. The detection of hypermethylation prior to NEC onset may serve as a potential biomarker for the detection of NEC. Moreover, it is possible that other exposures or medications that premature infants receive could alter the methylation patterns, but additional studies are necessary to answer these questions. Functional analysis of our data using IPA software revealed a number of results that both fit with our current understanding of NEC pathobiology and also demonstrate interesting novel themes which deserve further investigation. These analyses focused on both promoter DMRs and DMSs. The latter approach is designed to capture the broadest possible functional insight. It has the advantage of including single CpG sites that may exist in highly differentially methylated and disease-specific states. If a promoter contains one such site, then it is included in the analysis. In contrast, the use of promoter DMRs between sNEC and non-NEC samples likely captures more subtle effects that may be contributed by multiple CpG sites. In this study, with the goal of focusing on robust biological effects, we only included promoters with > 3 informative CpG sites and an average methylation difference of > 10%.

Given the likely significance of the microbiome on the development of NEC [21], it is notable that colonic sNEC-specific promoter DMRs are enriched for genes within the pathway, “role of pattern recognition receptors in the recognition of bacteria and viruses.” A number of genes in our DMR promoter data encode proteins previously shown to be associated with NEC pathobiology including TLR4 [12], PRKCZ [22], IL18 [23], IL17A [24]. Other genes shown in Additional file 1: Table S3, however, have not yet been associated with NEC, and these are worthy of further investigation. We also discovered that genes containing promoter DMRs were enriched for the “leukocyte extravasation signaling” and “interferon signaling” pathways, which suggests a role for epigenomic regulation of inflammatory processes in the colon affected by NEC. Differences in methylation signatures between sNEC and non-NEC ileum samples were less pronounced but still revealed enrichment for genes within pathways that are aligned with our emerging understanding of the pathobiology of NEC.

Notably, we demonstrated that the most highly significant predicted upstream regulator of the identified genes containing differentially methylated promoters in colon (DMR and DSS analyses) was HNF4A, a nuclear receptor transcription factor that regulates genes involved in intestinal epithelial cell development and function [25]. Accordingly, HNF4A RNA expression levels were also downregulated in our sNEC ileum samples as well as others [22]. HNF4A has also been shown to be important in the pathogenesis of inflammatory bowel disease as variants in human *HNF4A* are associated with increased risk of disease [26–28]. Taken together with our findings, this suggests not only that the expression of the transcriptional regulator HNF4A is significantly altered by the development of sNEC, but also that its gene regulatory activity may be influenced via epigenomic mechanisms.

More focused exploration of functional themes associated with promoter DMRs (Additional file 1: Table S2B) revealed some significant findings. For example, the GPR35 promoter was found to be the most hypermethylated promoter in sNEC colon compared to non-NEC colon. GPR35 is highly expressed in immune and intestinal epithelial cells, and its loss of function has been shown to promote colitis in an experimental animal model [29]. Similarly, the third most hypermethylated promoter in our data when comparing sNEC colon to control is located upstream of ring finger protein 186 (RNF186), which maintains gut homeostasis by controlling ER stress in colonic epithelium [30]. Furthermore, RNF186 gene variants have been shown to be associated with ulcerative colitis in humans. Other examples of promoters upstream of gut-associated genes that may advance our understanding of NEC pathobiology include, CDK5RAP3, which is known to act as a tumor suppressor in gastric cancer [31], PAK1, which is known to contribute to inflammatory conditions of the gastrointestinal tract including colitis [17, 32] and NR5A2, which is believed to contribute to the pathogenesis of inflammatory bowel disease [33–35].

In a similar fashion, we identified a number of functionally significant genes in which average promoter CpG methylation was significantly hypomethylated in sNEC colon versus non-NEC colon. Examples include, SOX9, an important regulator of cell proliferation in the intestinal epithelium [36, 37] and PDE4D, which is known to be associated with ulcerative colitis [38]. Furthermore, the loss of DOK2 has been shown to cause severe colitis in an animal model [39] and is altered at the level of DNA methylation in ulcerative colitis and Crohn’s disease in humans [40]. CD244 is a marker of intestinal immune cells, contributing to the protection against enteric pathogens and whose expression is dependent on the presence of the gut microbiota [41]. However, the role that CD224 plays in the context of epithelial cell signaling requires further investigation.

Our study has limitations relative to sample procurement and availability of clinical data. Due to the nature of one of our IRB protocols, a waiver of consent precluded us from obtaining clinical data from these infants, including the corrected age of the infant at the time of surgery. Infants with surgical NEC at the University of Pittsburgh typically have their reanastomosis surgery performed when they are near term-corrected age [42]. We acknowledge that these samples may have a component of intestinal adaptation that occurs after a bowel resection. However, it is a rare event that healthy term infants undergo bowel resection or intestinal biopsies and we have tried to capture as many non-NEC pathologies that are available in our biorepository. Thus, it is important to note that control tissue samples are in fact “healed NEC” tissue, obtained during surgical reanastomosis. These samples are the only available source of human tissue for this study design, and the practice of using these samples as non-NEC comparison tissues is common in NEC research. Furthermore, because of the manner in which samples were obtained and the nature of the de-identified tissue acquired, we were not able to obtain complete information regarding neonatal sex for all samples. We have therefore focused only on autosomal data to avoid the complication of X-inactivation to minimize the impact of sex differences.

In summary, we present the first systematic analysis of sNEC-specific DNA methylation changes in human infant gut epithelium. In addition to providing important reference data for future research, we have identified numerous genes and pathways that may be dysregulated at the level of the epigenome in sNEC. The data we present are comprehensive and hypothesis generating and will catalyze further investigation into epigenomic dysregulation and its consequences and potential therapeutic significance in NEC. Furthermore, given the rapid onset of NEC and its silent progression, there is an urgent need to identify biomarkers of NEC that may have utility for identifying at-risk individuals.

## Methods

All authors had access to the study data and had reviewed and approved the final manuscript.

### Declaration: ethics approval for study population and selection criteria

Intestinal samples were obtained from this study in accordance with the University of Pittsburgh anatomical tissue procurement guidelines and was approved by the University of Pittsburgh Institutional Review Board (IRB) Protocols (PRO09110437 or PRO14070508). Premature infants were recruited under Protocol PRO09110437 at either Children’s Hospital of Pittsburgh (CHP) of University of Pittsburgh Medical Center (UPMC) or Magee-Womens Hospital Neonatal Intensive Care Units (NICUs), and consent was obtained by a parent or legal guardian on behalf of their infant. Sample procurement included intestinal tissue if resected for NEC or other non-inflammatory indications such as reanastomosis, spontaneous intestinal perforation or anorectal malformation. In some instances, deidentified intestinal samples were obtained with a waiver of consent and approval of University of Pittsburgh IRB (PRO14070508). In these cases, the only clinical information able to be obtained was location of the resected intestine and the indication for surgery. Intestinal resections were snap frozen and stored at – 80 °C until laser capture microscopy.

### Laser capture microscopy

Snap-frozen specimens were mounted on appropriate embedding molds (Large, Thermo Scientific # 2219; or Small, Sakura Tissue—Tek # 4566) with clear OCT compound (Optimal Cutting Temperature Embedding Medium) (Fisher HealthCare # 4585) and sectioned on a cryostat instrument (Leica CM 1850 UV), 7 microns). These sections were mounted on membrane slides (Leica PEN—Membrane Slide, 2,0 microns # 11505158), stained with toluidine blue (toluidine blue 0.1% Aqueous, Newcomer Supply #14027) and air-dried (Sanpla Dry Keeper, Sanplatec. Corp). Laser dissection and capture of the chosen tissue region of interest (ROI) were cut by the laser capture microdissection (LCM) Instrument (Leica LMD 7000 microscope operated by the LMD software V7.3). The ROI laser capture tissue samples were subsequently collected into appropriate PCR tubes (Nushbaum, Inc. Large #110614-247 or small 110823-383) for further DNA extraction and subsequent studies.

### Whole genome bisulfite sequencing (WGBS)

DNA was extracted from LCM tissue using the Nucleospin Tissue XS Kit (Macherey–Nagel) with the modifications suggested for LCM. Extracted DNA was quantified by using the KAPA hgDNA Quantification and QC Kit (Roche). DNA was sheared with Covaris to a size of ~ 175 bp. WGBS libraries were prepared using the KAPA HyperPrep Kit (Roche). Libraries were bisulfite converted postligation using the EZ DNA Methylation-Direct Kit (Zymo). Post bisulfite conversion, libraries were amplified 12 cycles. Libraries were sequenced on an Illumina HiSeq using 150-bp paired-end reads. DNA sequence reads were quality trimmed, and adaptor sequences were removed using Trim-Galore (https://www.bioinformatics.babraham.ac.uk/projects/trim_galore/). The reads were aligned to the human reference sequence (GRCh38/hg38) using Bismark in paired-end Bowtie 2 modes. Unaligned paired-end reads were then processed in single-end mode. Read duplicates were removed using Bismark. Methylation was called on paired-end and single-end files and then merged. Differentially methylated sites were confirmed via multiplex PCR and next-generation sequencing of bisulfite converted DNA as previously described [14].

### RNA sequencing

Bulk RNA sequencing was performed on ileum and colon samples by the Genome Technology Access Center (GTAC) at the Washington University School of Medicine in St. Louis. Total RNA was isolated using TRIzol (Thermo Fisher). Ribosomal RNA was removed from 1μg of total RNA with RiboErase (KAPA). mRNA was then fragmented and reverse transcribed to yield cDNA using SuperScript III RT enzyme (Life Technologies, per manufacturer’s instructions) and random hexamers. A second-strand reaction was performed to yield ds-cDNA and Illumina sequencing adapters ligated to the ends. Ligated fragments were then amplified for 14 cycles using primers incorporating unique index tags. Fragments were sequenced on an Illumina NovaSeq using paired end reads extending 150 bases to a target of 30 M reads. Basecalls and demultiplexing were performed with Illumina’s bcl2fastq software and a custom python demultiplexing program with a maximum of one mismatch in the indexing read. mRNA expression was analyzed using Partek Flow software. Adapters were removed and reads were aligned to Genome Reference Consortium Human Build 38 with Bowtie 2 and quantified to Ensembl Transcripts release 96 with an average coverage of 31 and depth of 13. Reads were normalized to total counts per million. Features containing fewer than 10 total normalized reads or a lowest average coverage of 1 across all samples were not included the analysis. Partek flow gene-specific analysis was performed using a multi-model approach based on limma trend, which uses an empirical Bayes method to estimate gene expression.

### Quantitative real-time PCR

Tissue was stored in RNA Later (Thermo Fisher), and RNA was extracted with TRIzol (Thermo Fisher). RNA was quantified with a NanoDrop Spectrophotometer (Thermo Fisher). cDNA was made using the QuantiTech Reverse Transcription Kit (QIAGEN) according to the manufacturer’s instructions. Quantitative real-time PCR (qRT-PCR) was performed using IQ SYBR Green Supermix and the CFX Connect™ Real-Time PCR Detection System (Bio-Rad). The expression of genes assessed by qRT-PCR was quantified relative to the housekeeping gene RPLP0.

### Data analysis

To identify CpG sites differentially methylated between NEC and control samples, we performed logistical regression with correction for overdispersion, as implemented in the R package methylKit, on the number of methylated cytosines and unmethylated cytosines reported by Bismark. For differentially methylated (CpG) region (DMR) analyses on gene promoters, we defined the promoter region of a gene from 1500-bp upstream to 500 downstream of the transcription start site of that gene. To get the promoter and gene body methylation rate of each gene, we averaged the methylation rates of all CpG sites located inside, respectively, in the promoter region or the gene body. We further applied logit transformation to the methylation rates and used the empirical Bayesian method to test the difference in the promoter/gene body logit-transformed methylation rate between the NEC samples and non-NEC samples.

For analysis of associations between RNA expression and DNA methylation, we used the median of ratios method implemented in the R package DESeq2 to normalize the gene counts in each RNA-seq library and then performed log transformation on the normalized gene counts. We selected genes with the most significant difference (unadjusted *p* value ≤ 0.05, difference in logit-transformed methylation rate ≥ 1) in the promoter methylation level between the NEC and non-NEC samples. Then for each gene, we calculated the Pearson’s correlation between the log2 normalized gene expression across the samples and the corresponding promoter methylation rates. The Student’s t test was used to test the significance of the Pearson correlation coefficients. The *p* values of the tests were then adjusted using the Benjamini and Hochberg’s method to control the false discovery rate. A similar analysis was performed on the genes with the most significant difference in the gene body methylation level between the NEC and non-NEC samples.

## Supplementary information

**Additional file 1.** Supplementary Data Tables.

**Additional file 2.** Supplementary Figures.

## Data Availability

All data relevant to the study are included in the article or uploaded as supplementary information. Supplemental Table information can be found at the following link: https://wustl.box.com/s/o6apb4t142uwgzbc8xfgem5n0poxiabi
