# Supplementary material for: Global hypermethylation of intestinal epithelial cells is a hallmark feature of neonatal surgical necrotizing enterocolitis
Source: Clin Epigenetics. 2020 Dec 11;12:190. doi: 10.1186/s13148-020-00983-6 (PMC7730811; doi:10.1186/s13148-020-00983-6)
Supplement: Supplementary file 2 — Additional file 2. Supplementary Figures. [file 13148_2020_983_MOESM2_ESM.zip › 13148_2020_983_MOESM2_ESM/FIGURE LEGENDS.docx]

**Additional file Figure Legends:**

**Figure S1:** Density plots of CpG methylation levels across the entire genome using WGBS data from NEC and non-NEC colon (A) or Ileum (B) tissue samples. Red lines represent NEC colon and blue lines represent non-NEC colon. These plots report, in each case, the global distribution of CpG methylation levels without reference to genomic location in; all sites, promoters, exons, introns, intergenic regions, CpG island shores, enhancers and CpG Islands (CGI).

**Figure S2:** CpG methylation levels mapped spatially across all autosomes. Red lines represent NEC ileum and blue lines represent non-NEC colon. Data are presented for (A) all sites, (B) promoters, (C) exons, (D) introns, (E) intergenic regions, (F) enhancers, (G) CpG island shores and (H) CpG islands (CGI).

**Figure S3:** Ingenuity pathway analysis (IPA) results for promoter differentially-methylated CpG regions (DMRs) between NEC colon and non-NEC colon. (A) Pathway diagram for “role of pattern recognition receptors in the recognition of bacteria and viruses” (p=8.44x10^-4^). (B-D) Heat maps of genes within IPA-identified enriched pathways, “role of pattern recognition receptors in the recognition of bacteria and viruses”, “leukocyte extravasation signaling” and “triacylglycerol biosynthesis” respectively. NEC and non-NEC samples are annotated in red and black respectively.

**Figure S4:** Ingenuity pathway analysis results for promoter DMRs between NEC colon and non-NEC colon. (A) “role of pattern recognition receptors in the recognition of bacteria and viruses” (p=8.44x10^-4^), (B) “leukocyte extravasation signaling” (p=1.28x10^-3^), (C) “triacylglycerol biosynthesis” (p=2.33x10^-3^) and (D) “interferon signaling (p=3.71x10^-3^).

**Figure S5:** (A) Predicted HNF4A-regulated genes and (B) HNF1A-regulated genes identified by IPA analysis of NEC-specific colon differentially-methylated CpG regions (DMRs). (C) Plot of percent methylation value for a given site (n=33) from WGBS data set (x-axis) versus follow-up multiplex amplicon sequencing (y-axis). Control colon is blue and NEC colon is red. The dashed line represents a correlation value of 1 between the two data sets. Actual Pearson correlation between the sequencing methods was 0.965 and 0.940 for control and NEC colon respectively.

**Figure S6:** Ingenuity pathway analysis results for promoter DMRs between NEC ileum and non-NEC ileum. (A) “granulocyte adhesion and diapedesis” (1.34x10^-2^), (B) “induction of apoptosis by HIV1” (1.35x10^-2^), (C) “IL-17 signaling” (1.69x10^-2^), and (D) “CD40 signaling” (1.81x10^-2^).

**Figure S7:** Ingenuity pathway analysis (IPA) results for promoter differentially methylated single CpG sites (DMSs) between NEC colon and non-NEC colon. (A) Pathway diagram for “integrin signaling” (p=2.32x10^-10^), (B-D) Heat maps of genes within IPA-identified enriched pathways, (B) “integrin signaling”, (C) “molecular mechanisms of cancer” and (D) “ERK/MAPK signaling” respectively. NEC and non-NEC samples are annotated in red and black respectively.

**Figure S8:** Ingenuity pathway analysis results for promoter DMSs between NEC colon and non-NEC colon. (A) “integrin signaling” (p=2.32x10^-10^), (B) “molecular mechanisms of cancer” (p=1.62x10^-8^), (C) “ERK/MAPK signaling” (p=1.21x10^-6^) and (D) “leukocyte extravasation signaling” (p=2.05x10^-6^).

**Figure S9:** Ingenuity pathway analysis (IPA) results for promoter differentially methylated single CpG sites (DMSs) between NEC colon and non-NEC colon. (A) Pathway diagram for “molecular mechanisms of cancer” (p=3.07 x 10^-7^), (B-D) Heat maps of genes within IPA-identified enriched pathways, (B) “molecular mechanisms of cancer”, (C) “AMPK signaling” and (D) PDGF signaling respectively. NEC and non-NEC samples are annotated in red and black respectively.

**Figure S10:** Ingenuity pathway analysis results for CpG island shore DMSs between NEC colon and non-NEC colon. (A) “molecular mechanisms of cancer” (p=3.07 x 10^-7^), (B) “AMPK signaling” (p=3.01x10^-5^), (C) PDGF signaling (p=9.58x^10-5^) and (D) “PPARα/RXRα activation” (p=1.6x10-4).

**Figure S11:** Functional pathway analysis, using IPA, of differentially expressed transcripts identified in colon (A-C) and ileum (D-F) revealed enrichment of genes in pathways for (A) “hepatic fibrosis/hepatic stellate activation” (p=3.68x10^-10^), (B) “granulocyte adhesion and diapedesis” (p=8.76x10^-9^)”, (C) “axonal guidance” (p=4.98x10^-8^), (D) “acute phase response signaling (p=3.84x10^-8^), (E) “granulocyte adhesion and diapedesis” (p=4.24x10^-8^) and (F) “hepatic fibrosis/hepatic stellate activation” (p=1.46x10^-6^).

**Figure S12:** Ingenuity pathway analysis results gene expression changes (for p=<0.05, least squares (LS) mean >1, 2-fold differential expression) between NEC colon and non-NEC colon. (A) “hepatic fibrosis/hepatic stellate activation” (p=3.68x10^-10^),(B) “granulocyte adhesion and diapedesis” (p=8.76x10^-9^) and (C) “axonal guidance” (p=4.98x10^-8^).

**Figure S13:** Ingenuity pathway analysis results gene expression changes (for p=<0.05, least squares (LS) mean >1) between NEC ileum and non-NEC ileum. (A) “acute phase response signaling” (p=3.84x10^-8^), (B) “granulocyte adhesion and diapedesis” (p=4.24x10^-8^) and (C) “hepatic fibrosis/hepatic stellate activation” (p=1.46x10^-6^).

**Figure S14:** Functional analysis using IPA of overlap between protein coding RNAs that are differentially expressed (p=<0.05) in NEC vs. non-NEC colon and the corresponding DMRs present in our NEC vs. non-NEC colon data. (A) endothelin 1 signaling (p=2.70x10^-4^), (B) corticotropin releasing hormone signaling (p=1.37x10^-3^) and (C) hepatic cholestasis (2.62x10^-3^).
