# Supplementary material for: Global hypermethylation of intestinal epithelial cells is a hallmark feature of neonatal surgical necrotizing enterocolitis
Source: Clin Epigenetics. 2020 Dec 11;12:190. doi: 10.1186/s13148-020-00983-6 (PMC7730811; doi:10.1186/s13148-020-00983-6)
Supplement: Supplementary file 2 — Additional file 2. Supplementary Figures. [file 13148_2020_983_MOESM2_ESM.zip › 13148_2020_983_MOESM2_ESM/Figures Supplemental Clinical Epigenetics FINAL.pptx]

## Slide 1
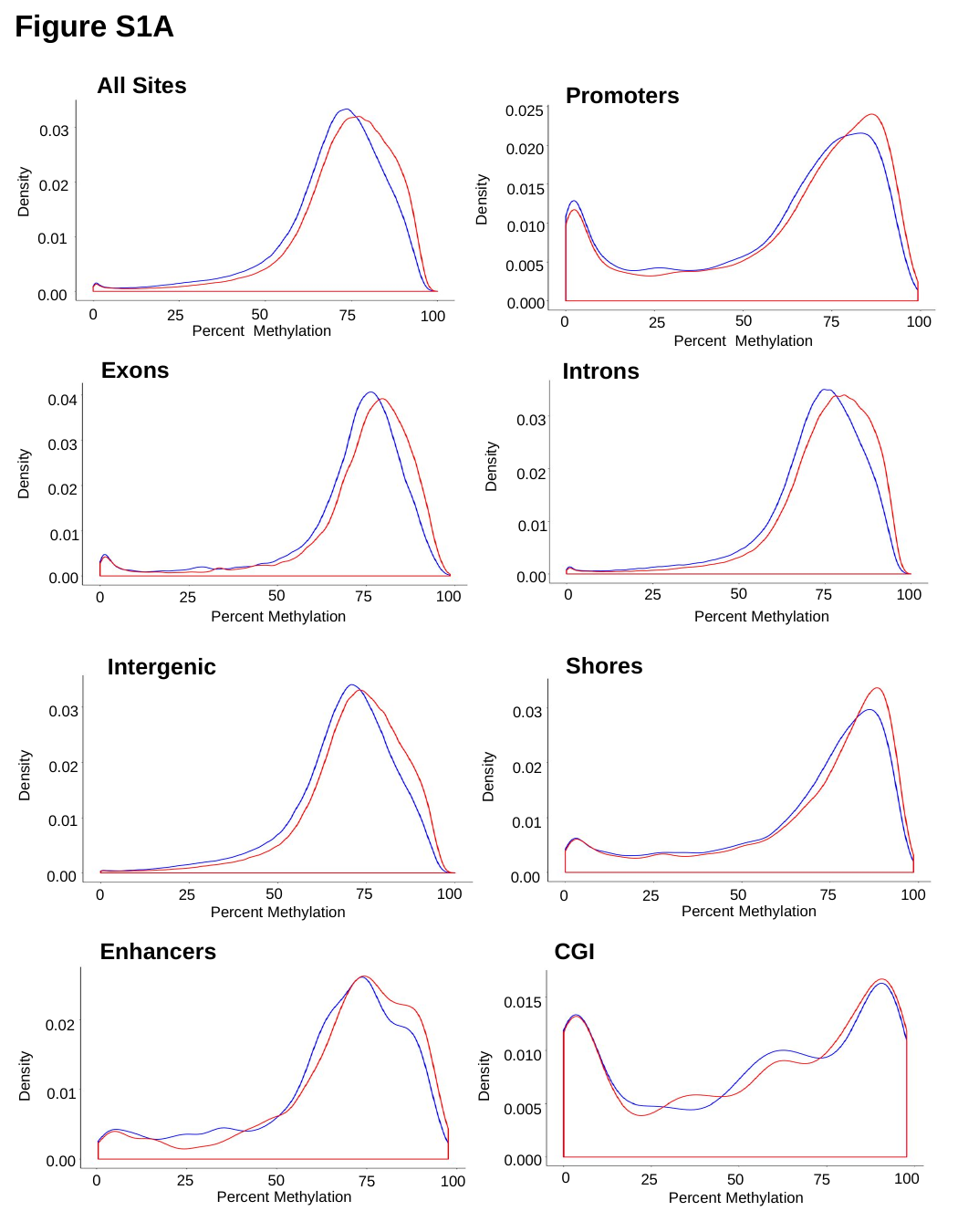

Figure S1A
All Sites
0.03
0.02
Density
0.01
0.00
 0
50
25
75
100
Percent Methylation
Promoters
0.025
0.020
0.015
Density
0.010
0.005
0.000
50
75
100
 0
25
Percent Methylation
Exons
0.04
0.03
Density
0.02
0.01
0.00
50
75
100
25
 0
Percent Methylation
Introns
0.03
Density
0.02
0.01
0.00
50
75
100
 0
25
Percent Methylation
Shores
0.03
0.02
Density
0.01
0.00
50
75
100
 0
25
Percent Methylation
Intergenic
0.03
0.02
Density
0.01
0.00
50
75
100
 0
25
Percent Methylation
Enhancers
0.02
Density
0.01
0.00
50
 0
25
100
75
Percent Methylation
CGI
0.015
0.010
Density
0.005
0.000
 0
100
25
75
50
Percent Methylation

## Slide 2
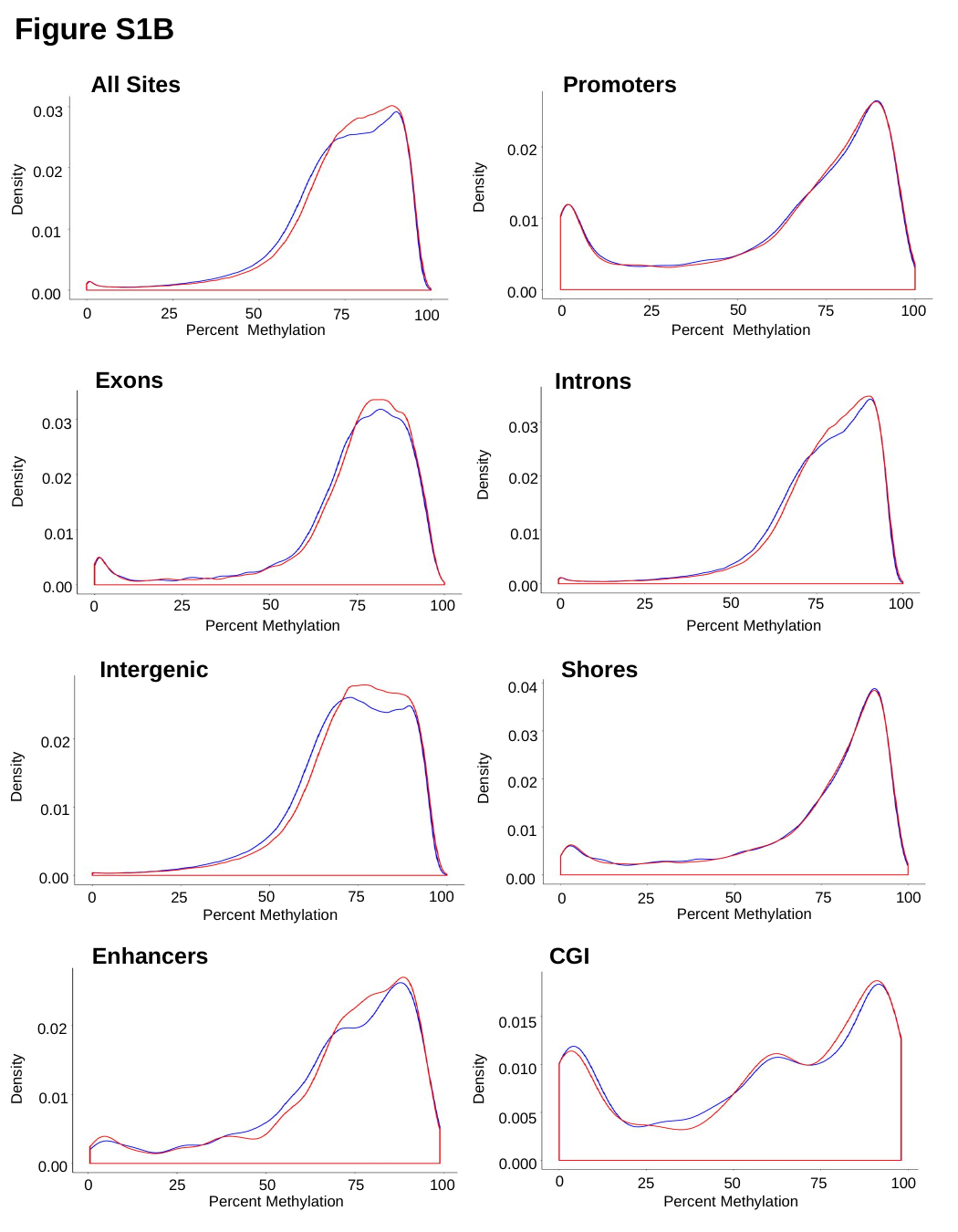

Figure S1B
Promoters
0.02
Density
0.01
0.00
50
75
100
 0
25
Percent Methylation
All Sites
0.03
0.02
Density
0.01
0.00
 0
50
25
75
100
Percent Methylation
Exons
0.03
0.02
Density
0.01
0.00
50
75
100
25
 0
Percent Methylation
Introns
0.03
0.02
Density
0.01
0.00
50
75
100
 0
25
Percent Methylation
Shores
0.04
0.03
0.02
Density
0.01
0.00
50
75
100
 0
25
Percent Methylation
Intergenic
0.02
Density
0.01
0.00
50
75
100
 0
25
Percent Methylation
Enhancers
0.02
Density
0.01
0.00
50
 0
25
100
75
Percent Methylation
CGI
0.015
0.010
Density
0.005
0.000
 0
100
25
75
50
Percent Methylation

## Slide 3
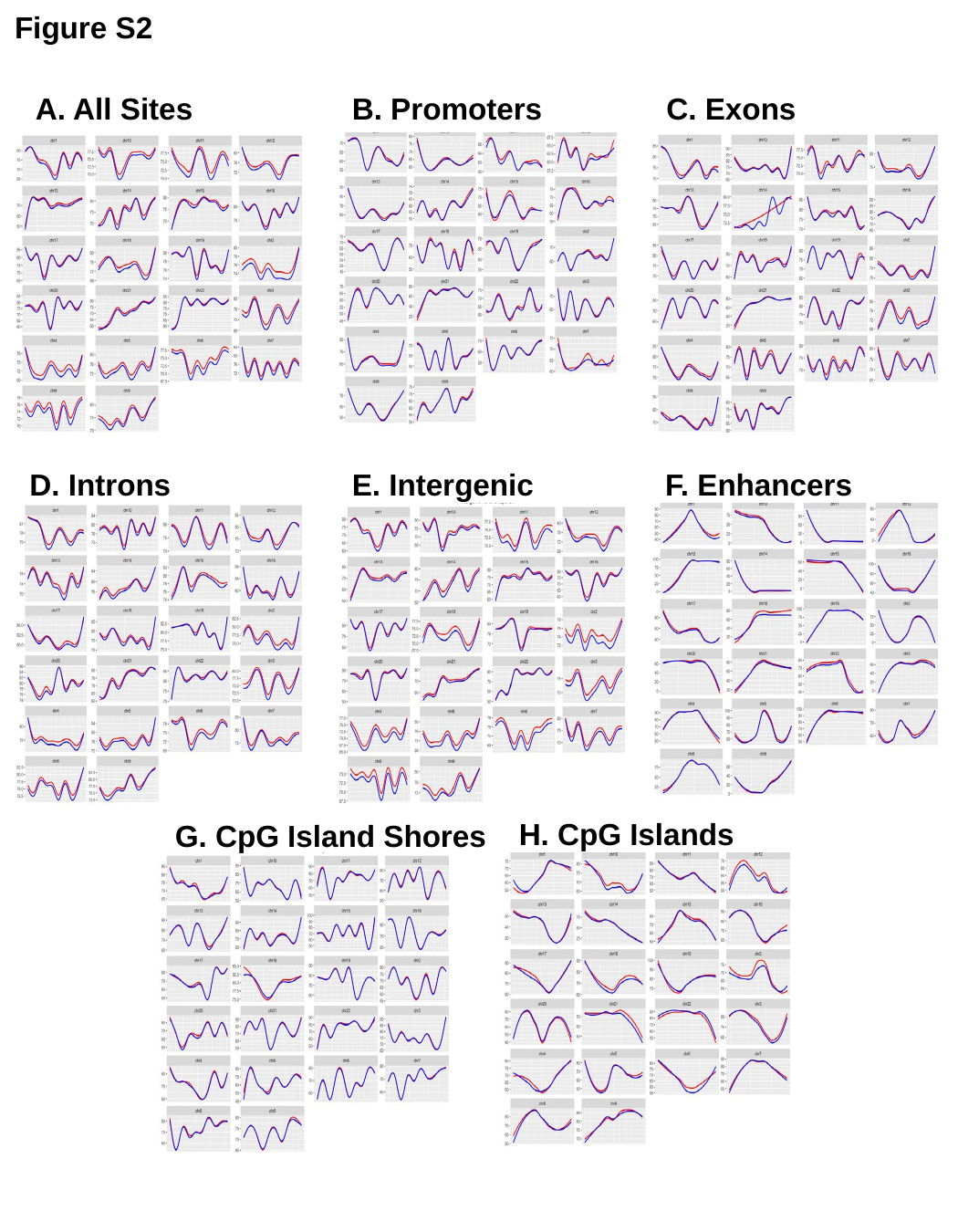

Figure S2
A. All Sites
B. Promoters
C. Exons
D. Introns
E. Intergenic
F. Enhancers
H. CpG Islands
G. CpG Island Shores

## Slide 4
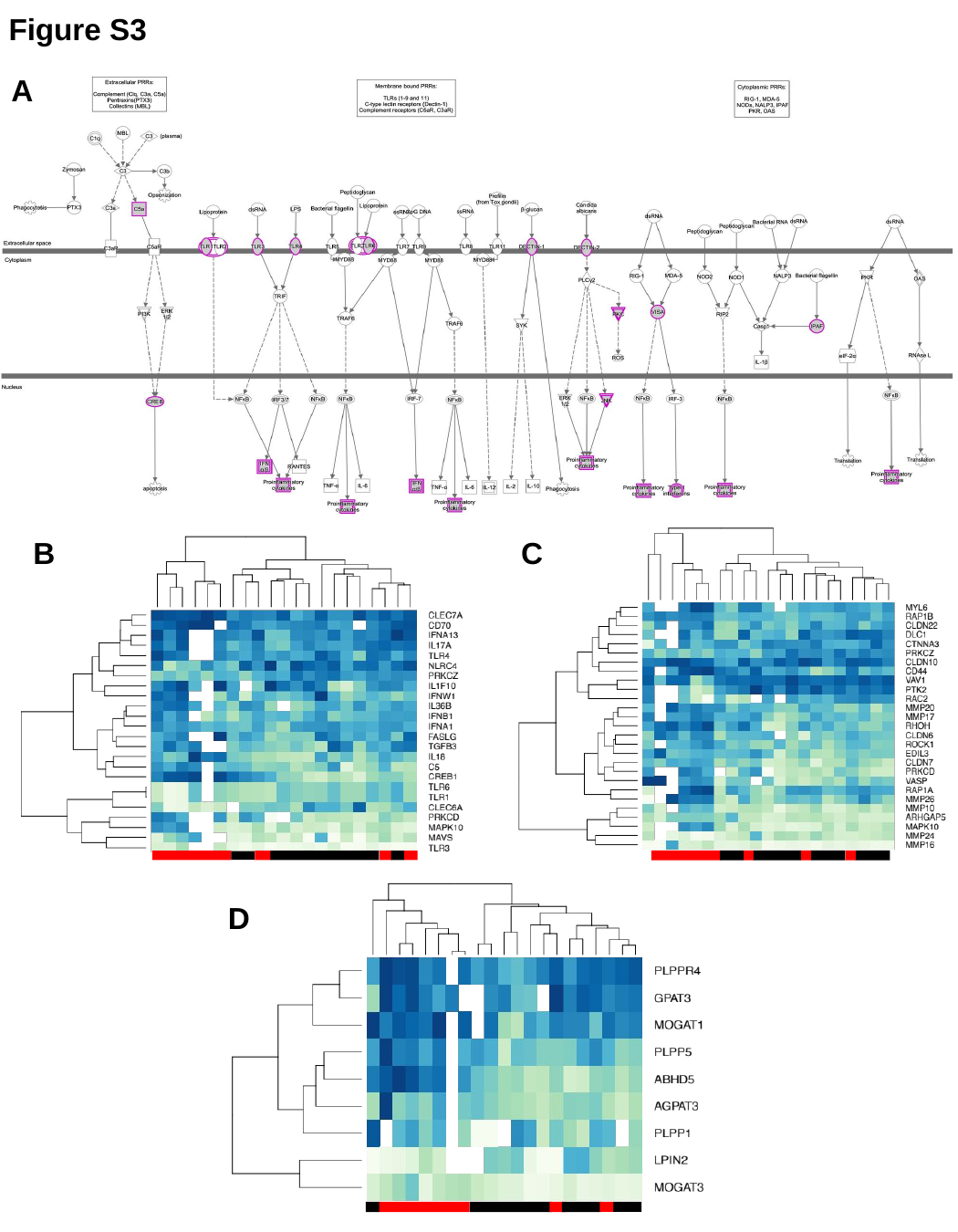

Figure S3
A
B
C
D

## Slide 5
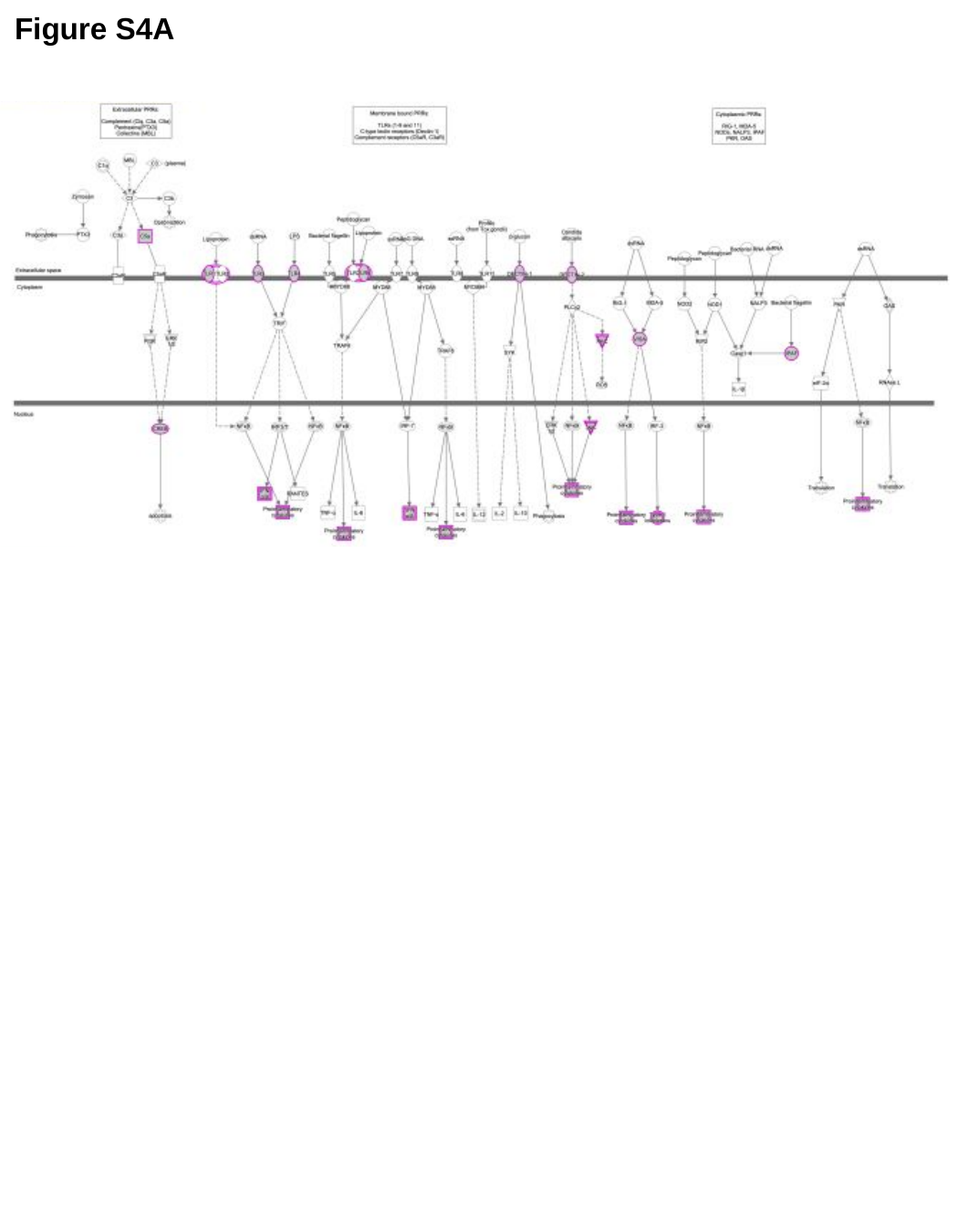

Figure S4A

## Slide 6
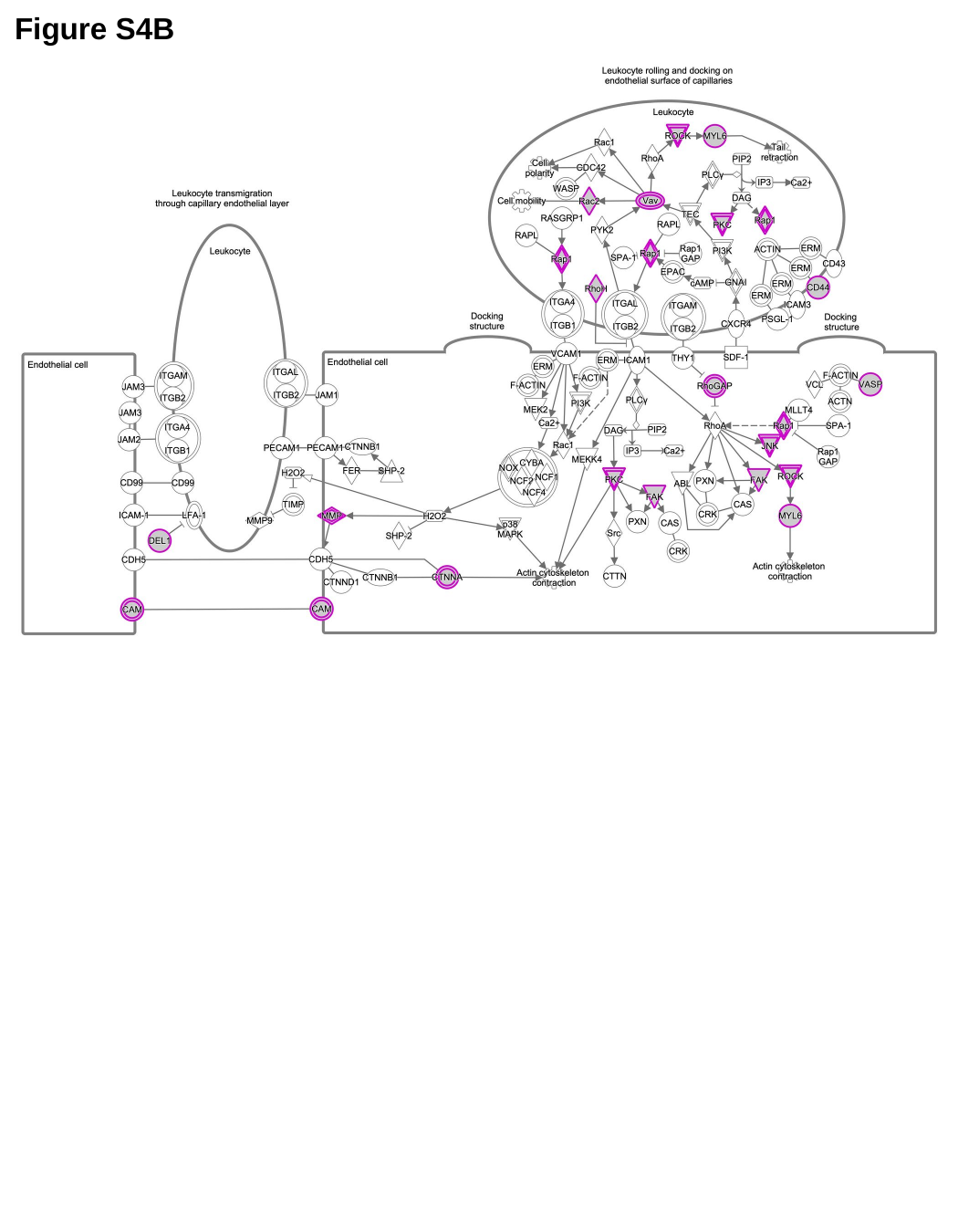

Figure S4B

## Slide 7
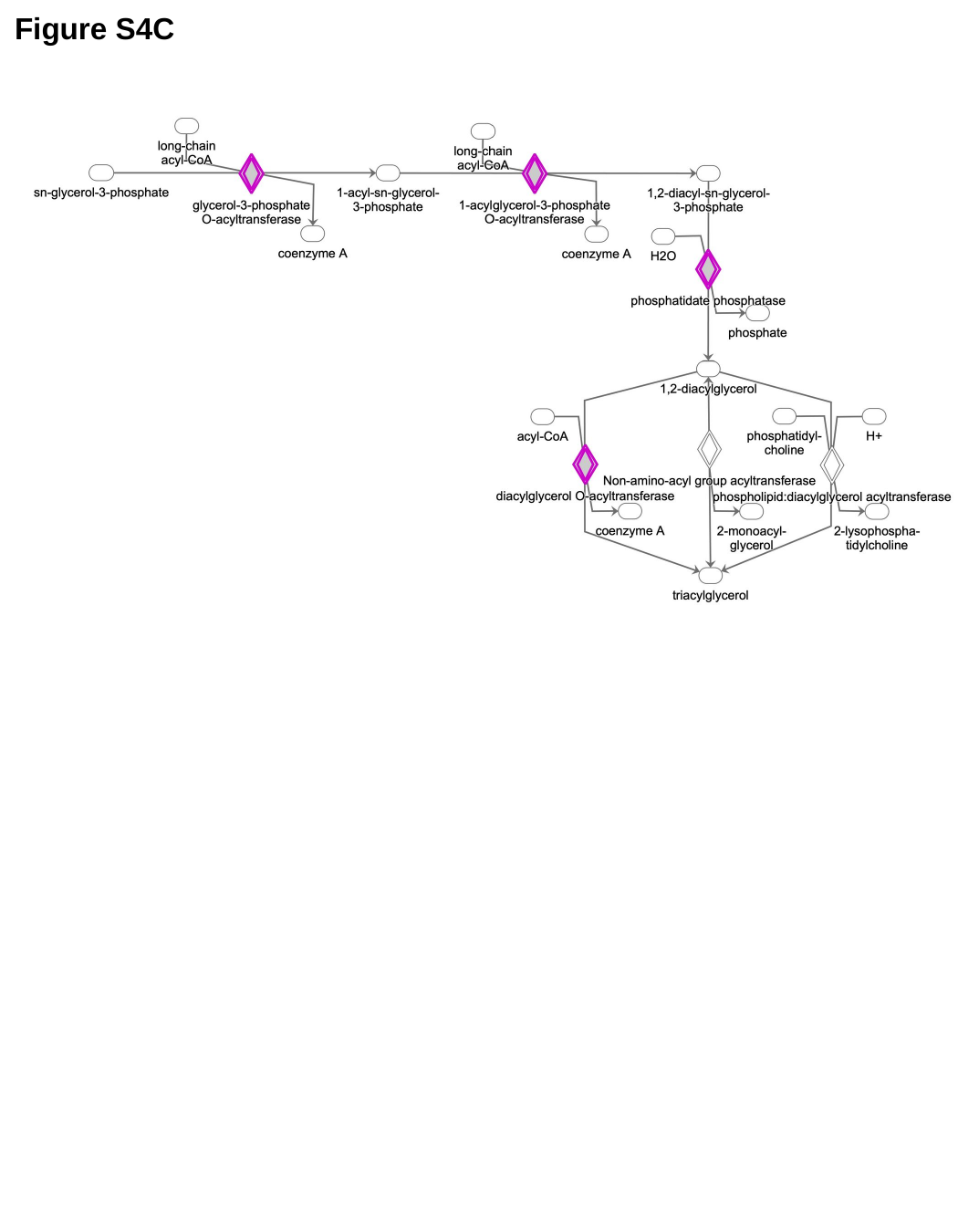

Figure S4C

## Slide 8
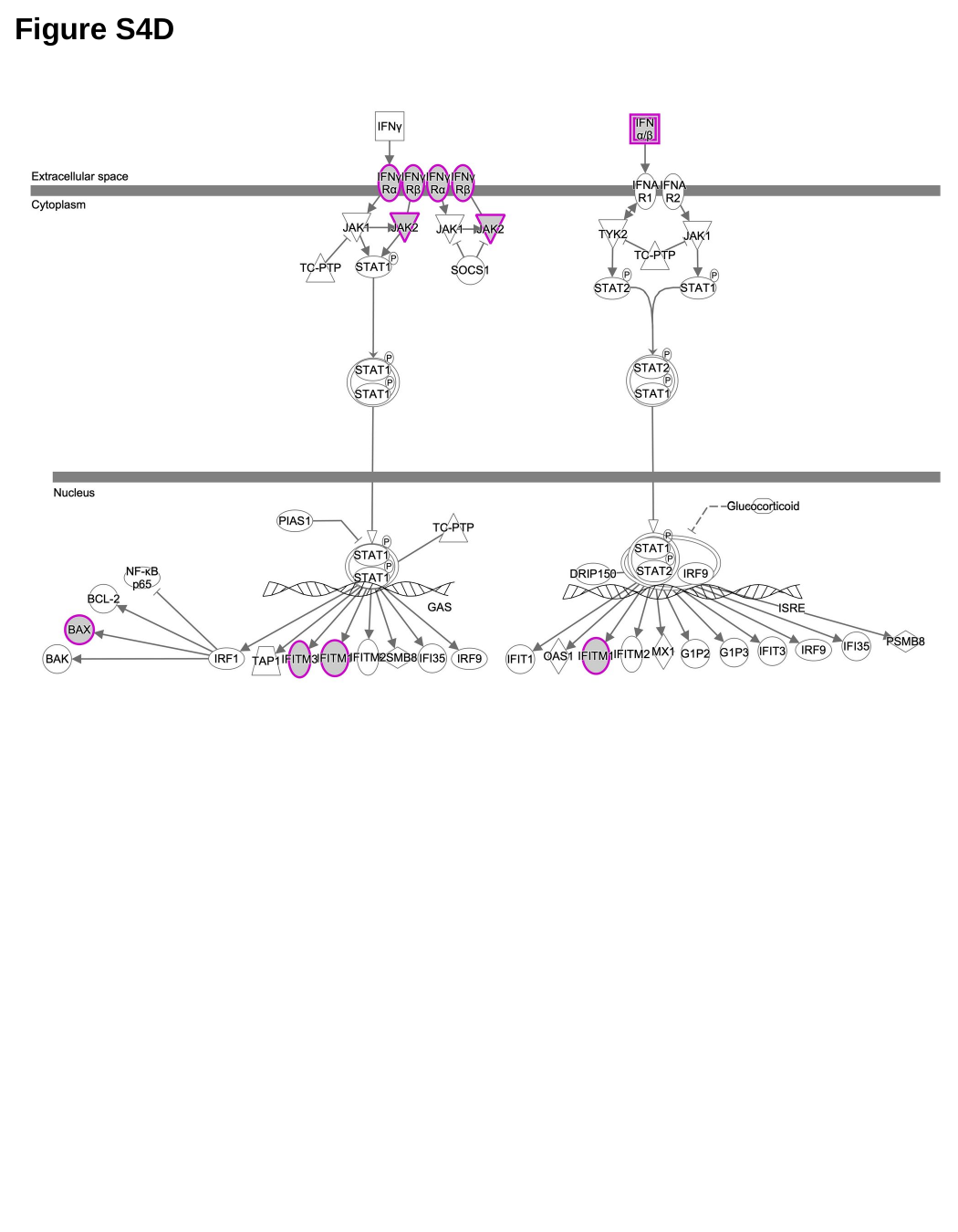

Figure S4D

## Slide 9
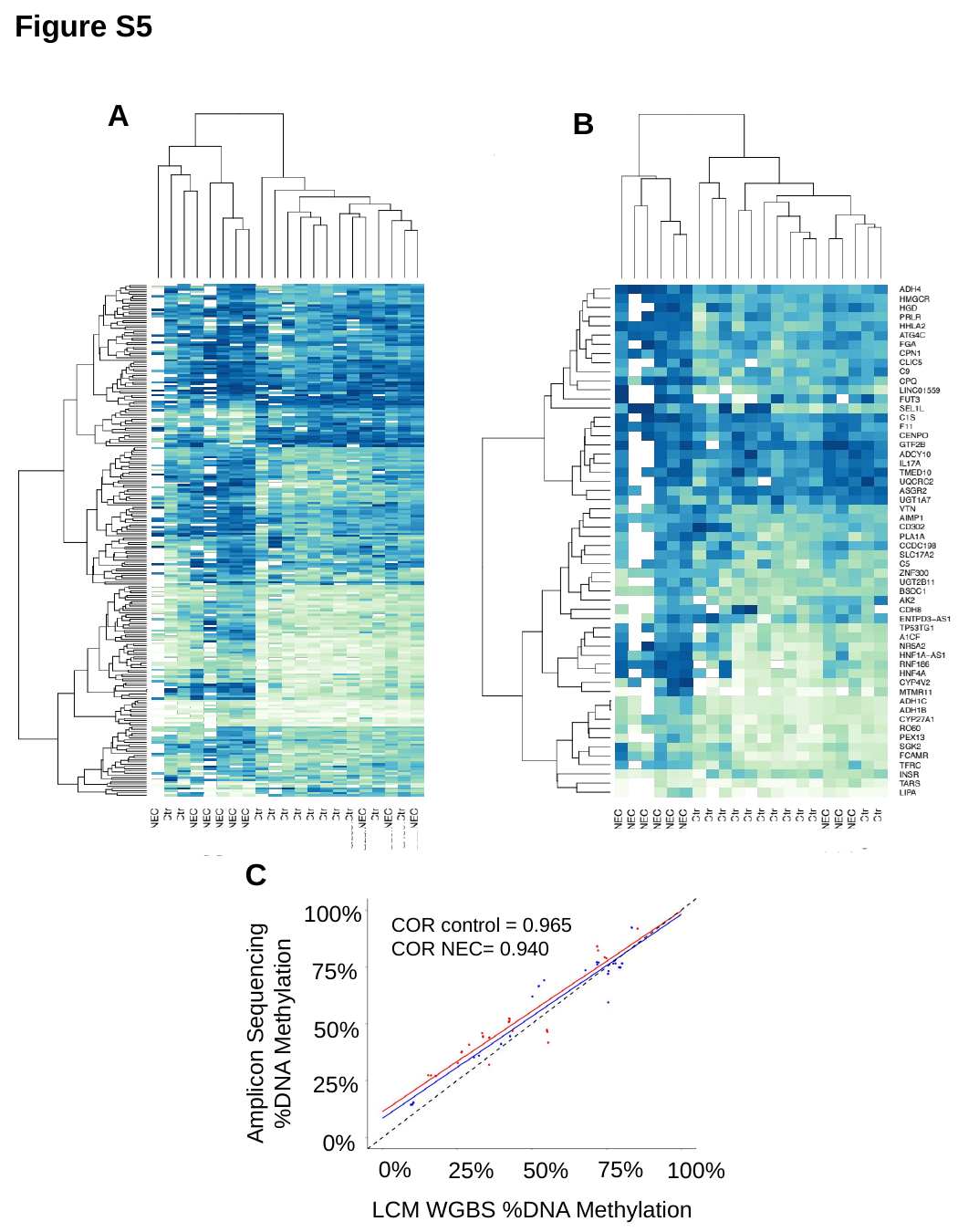

Figure S5
A
B
C
100%
COR control = 0.965
COR NEC= 0.940
75%
50%
25%
0%
75%
0%
25%
50%
100%
Amplicon Sequencing %DNA Methylation
LCM WGBS %DNA Methylation

## Slide 10
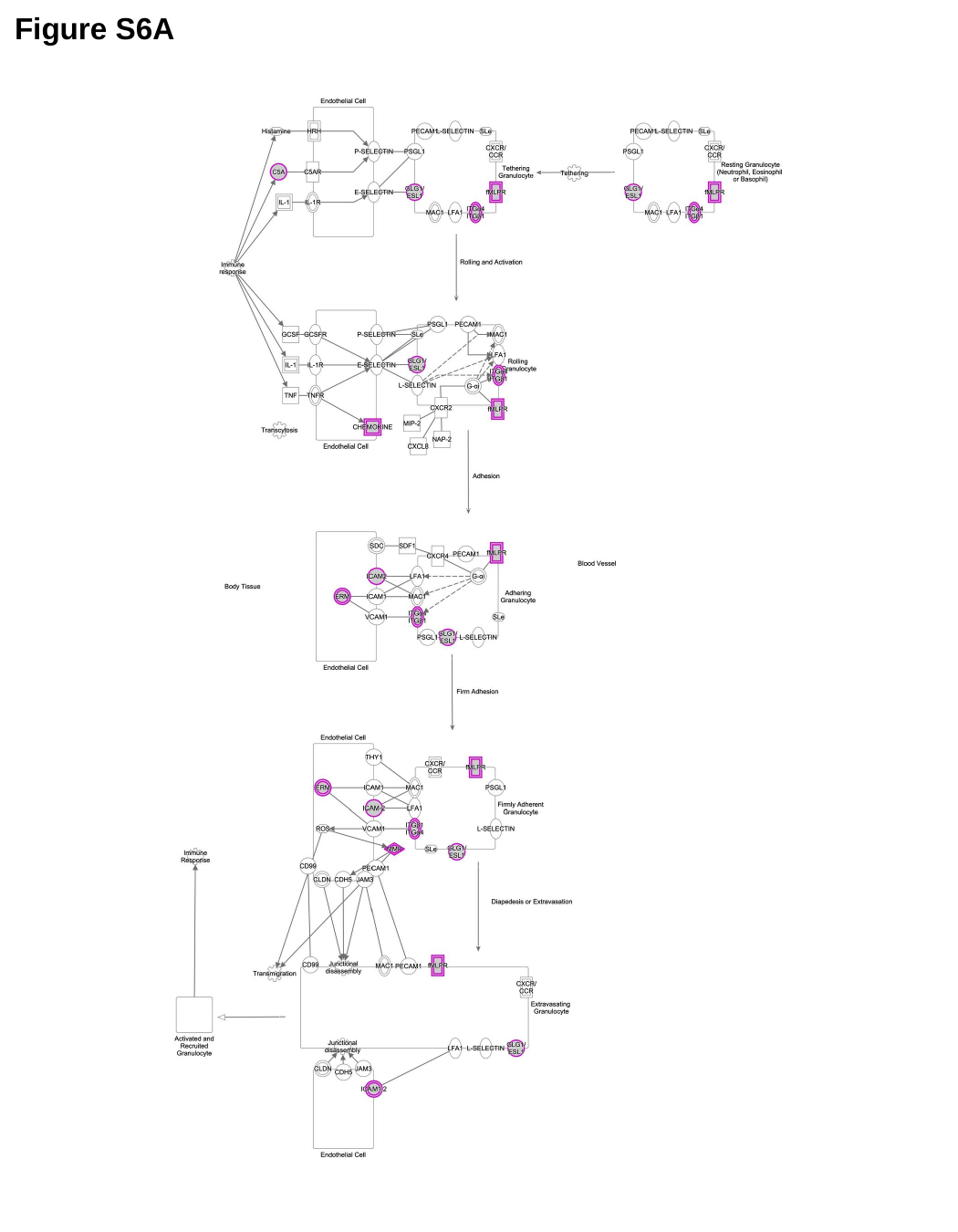

Figure S6A

## Slide 11
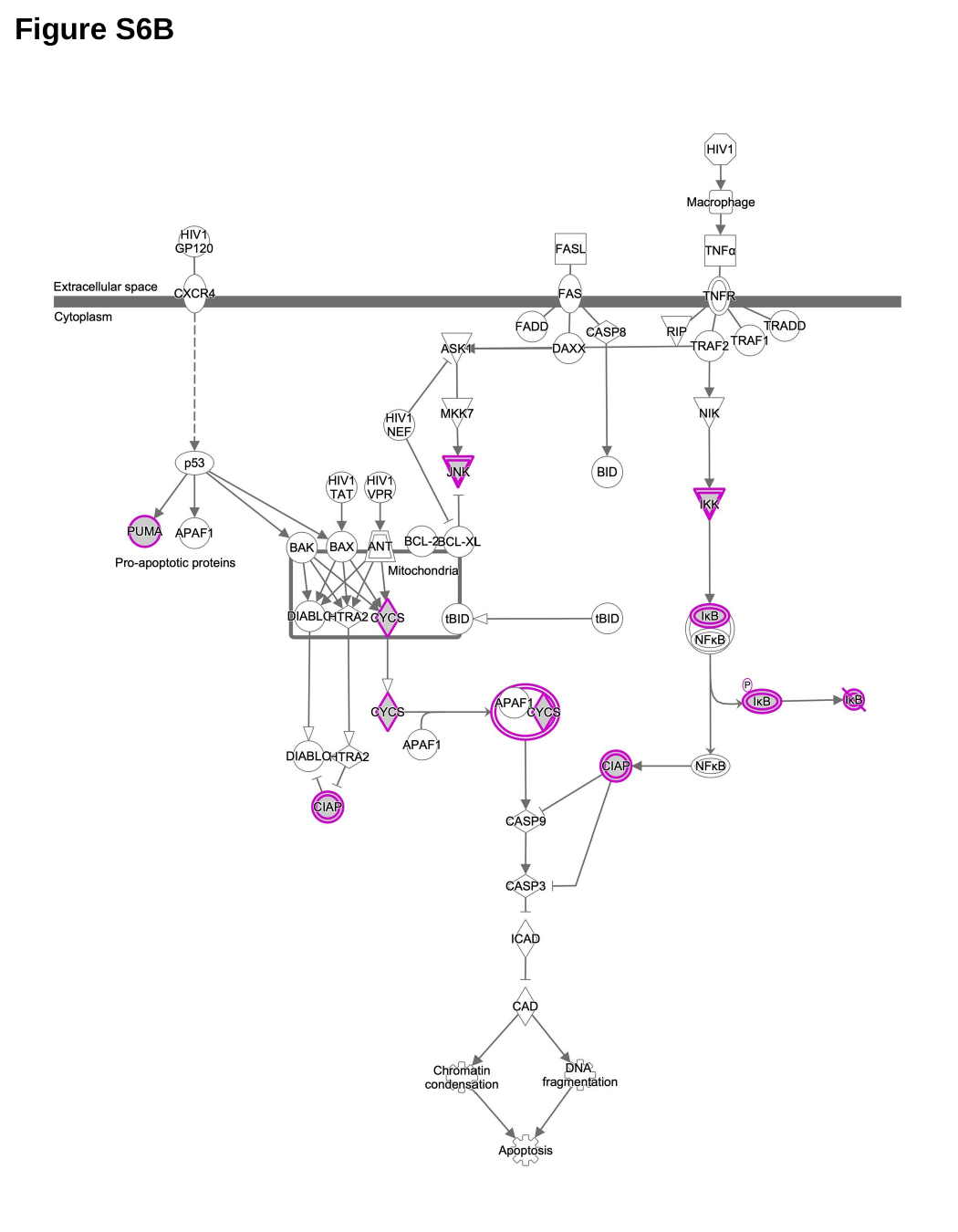

Figure S6B

## Slide 12
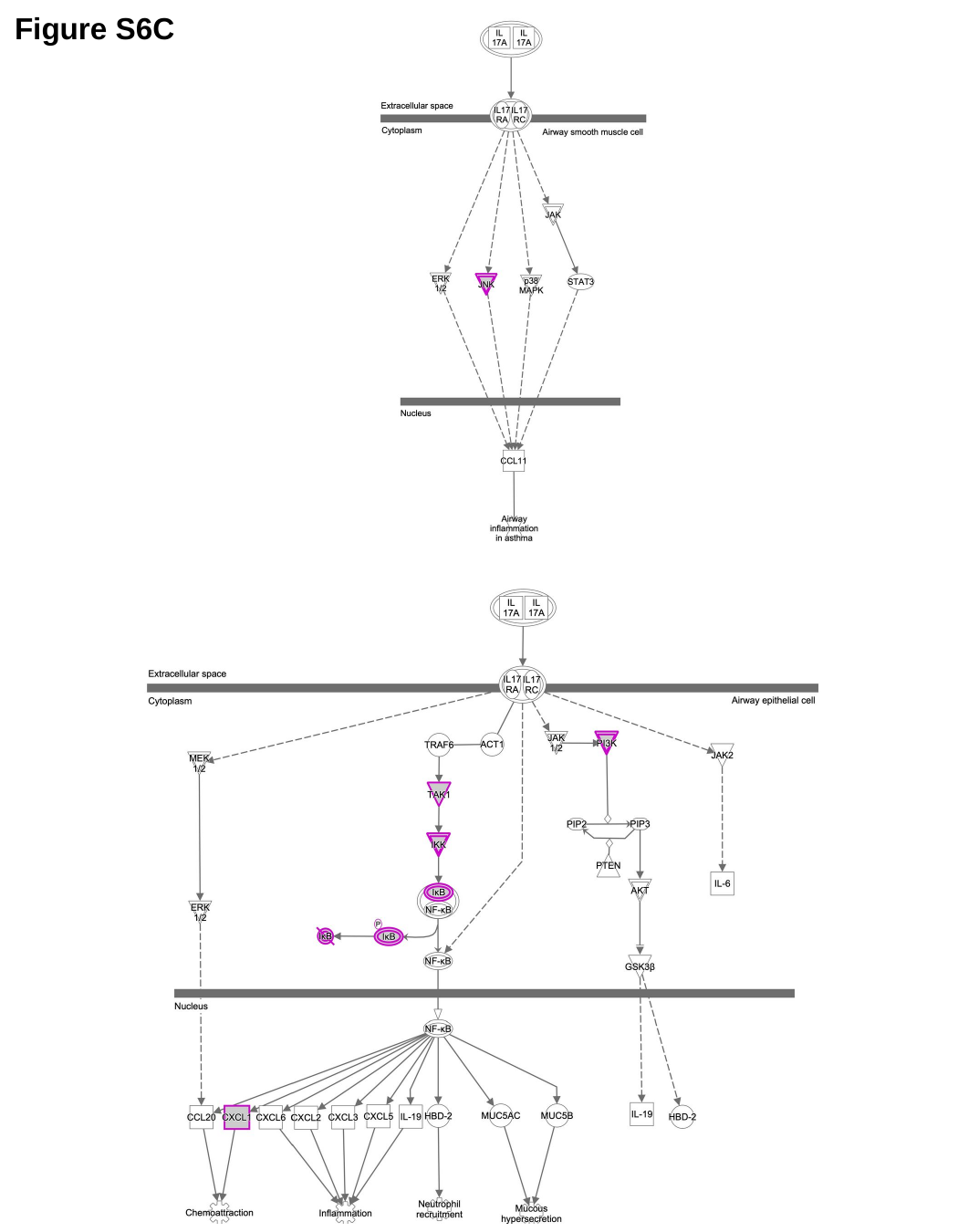

Figure S6C

## Slide 13
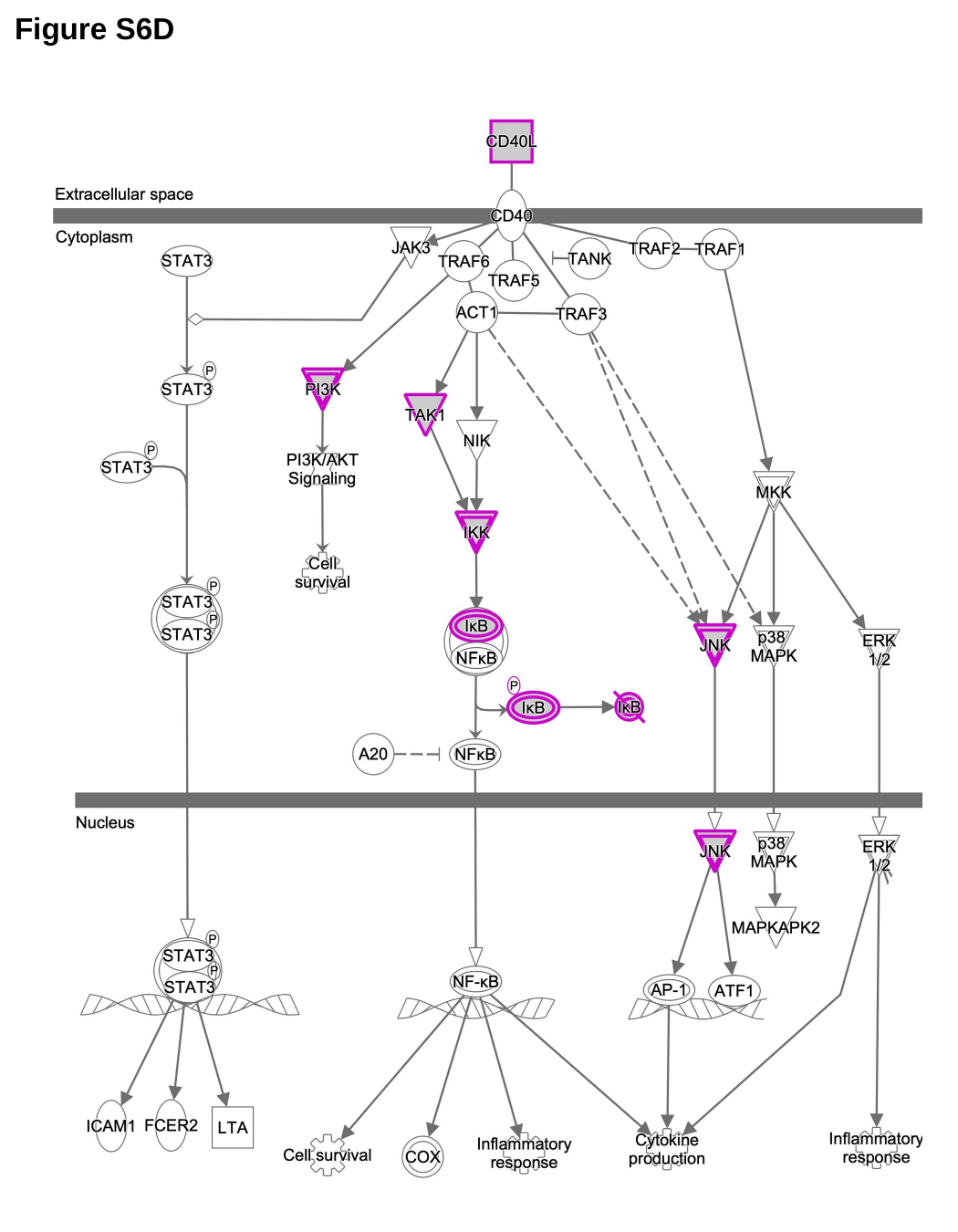

Figure S6D

## Slide 14
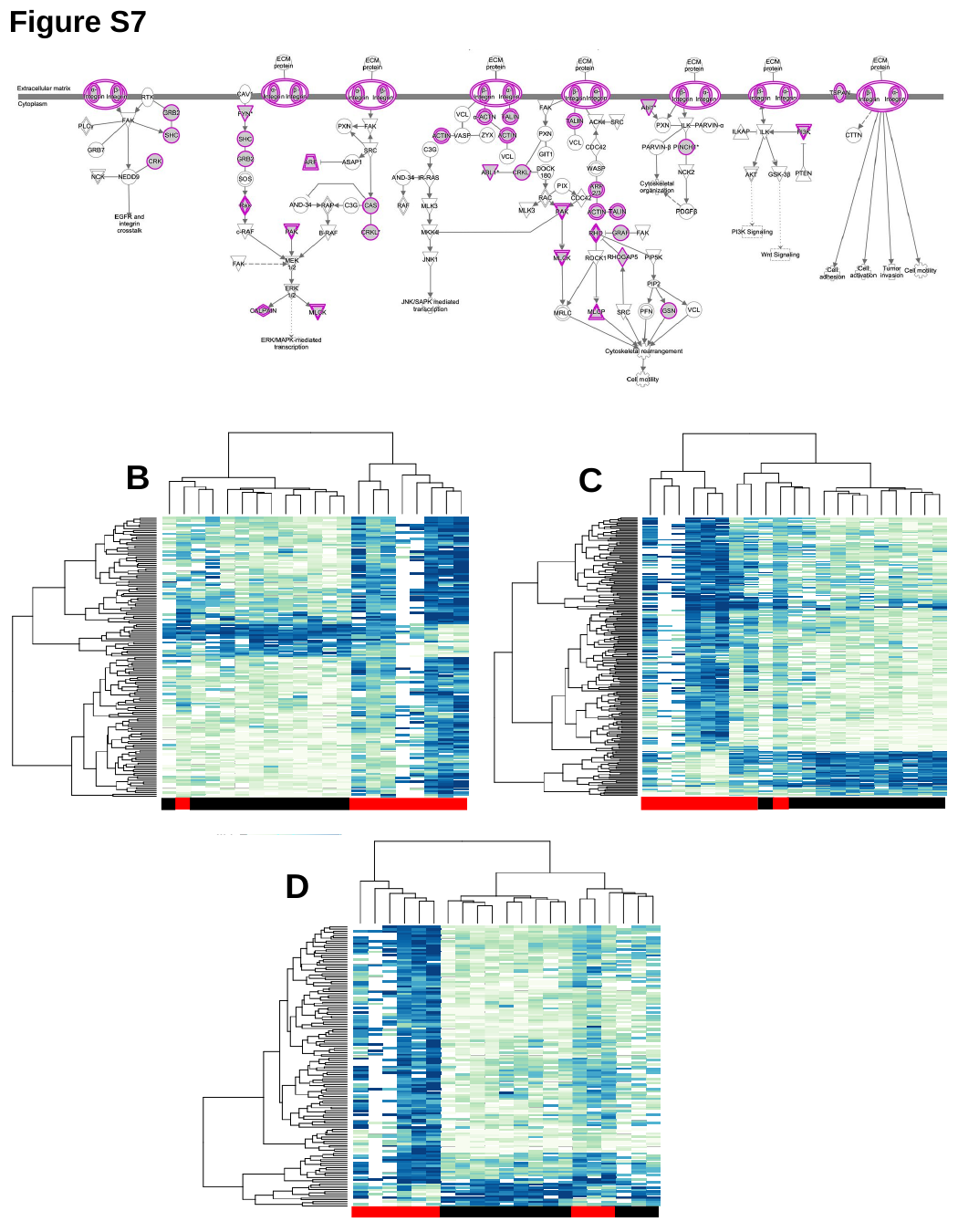

Figure S7
C
B
D

## Slide 15
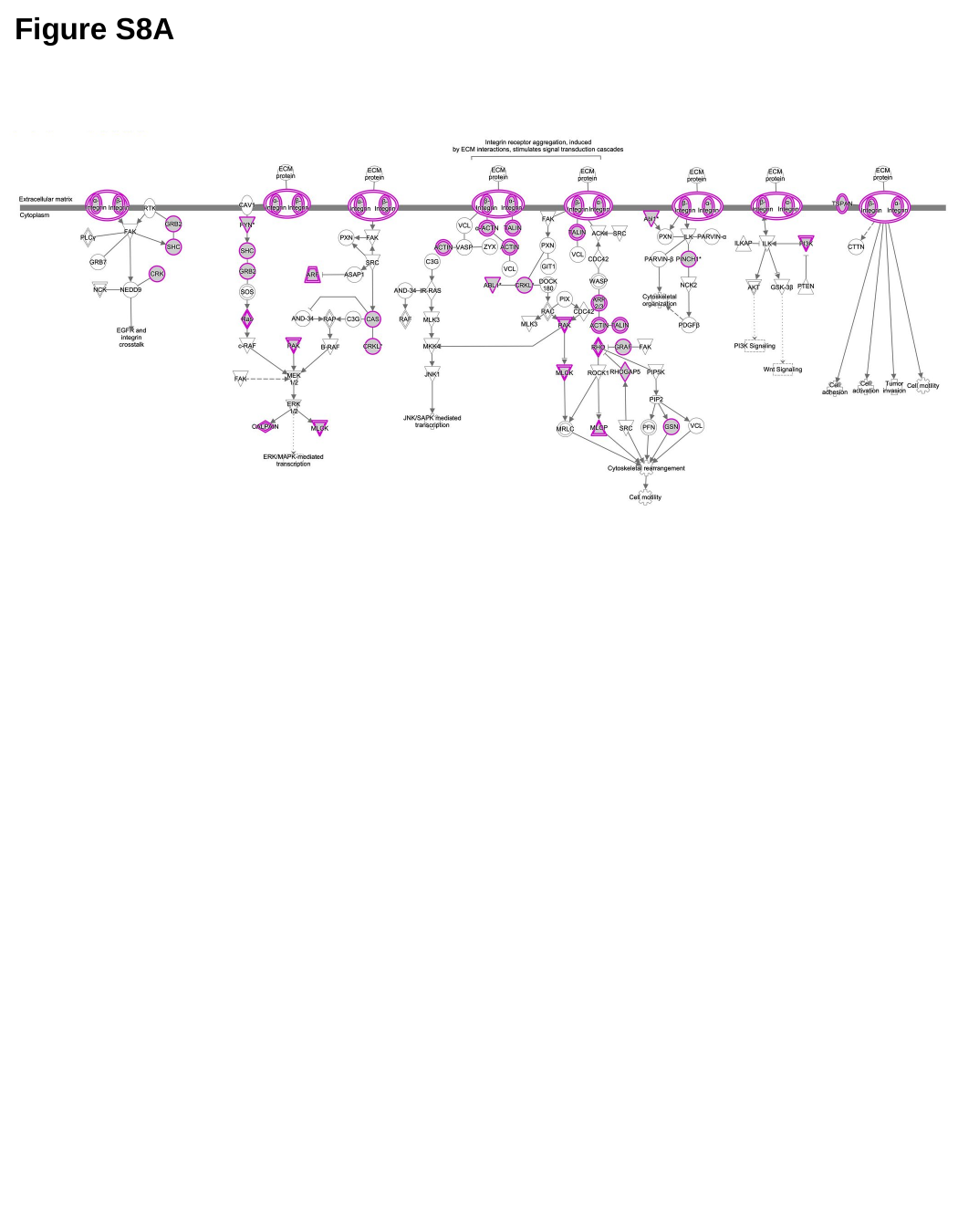

Figure S8A

## Slide 16
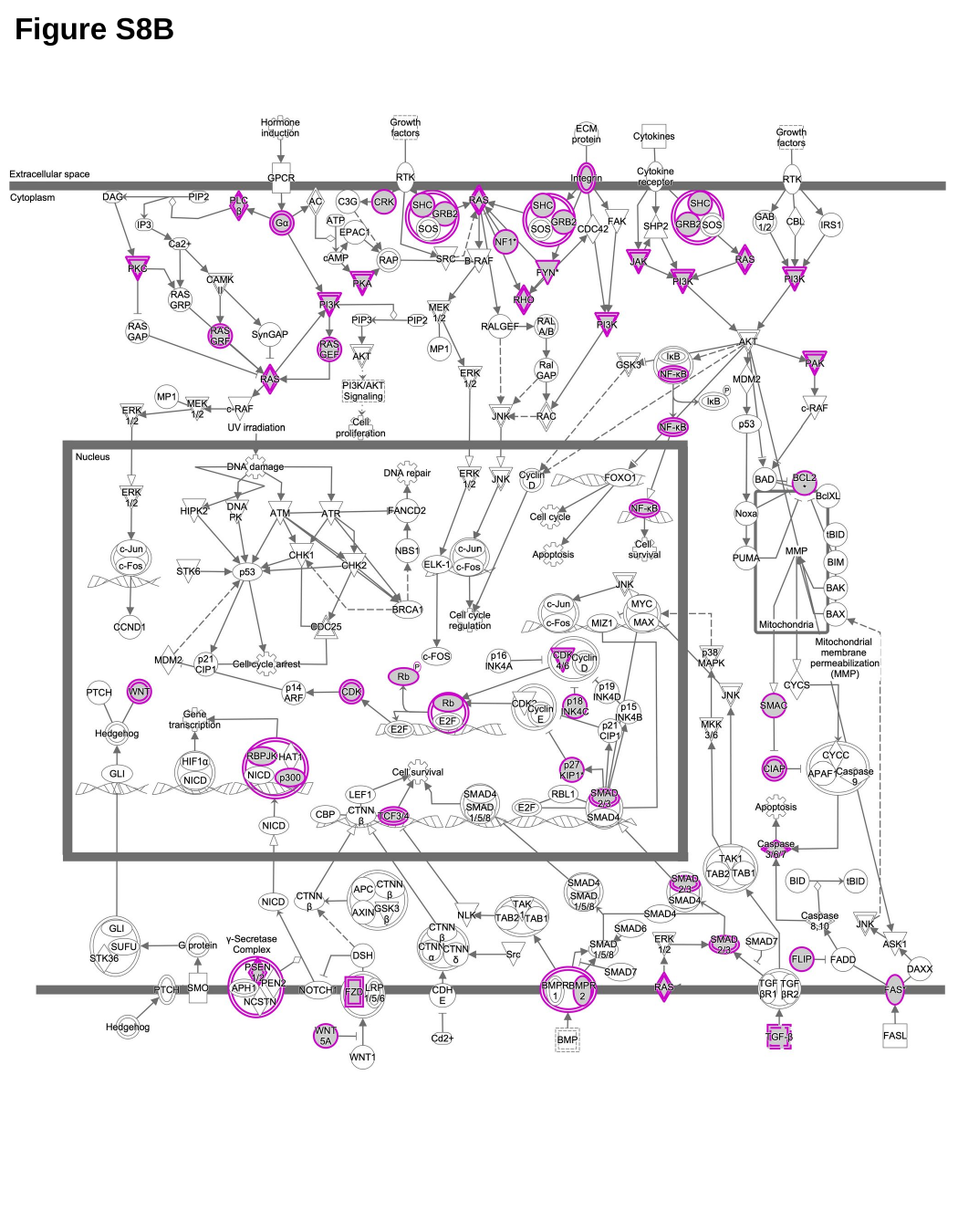

Figure S8B

## Slide 17
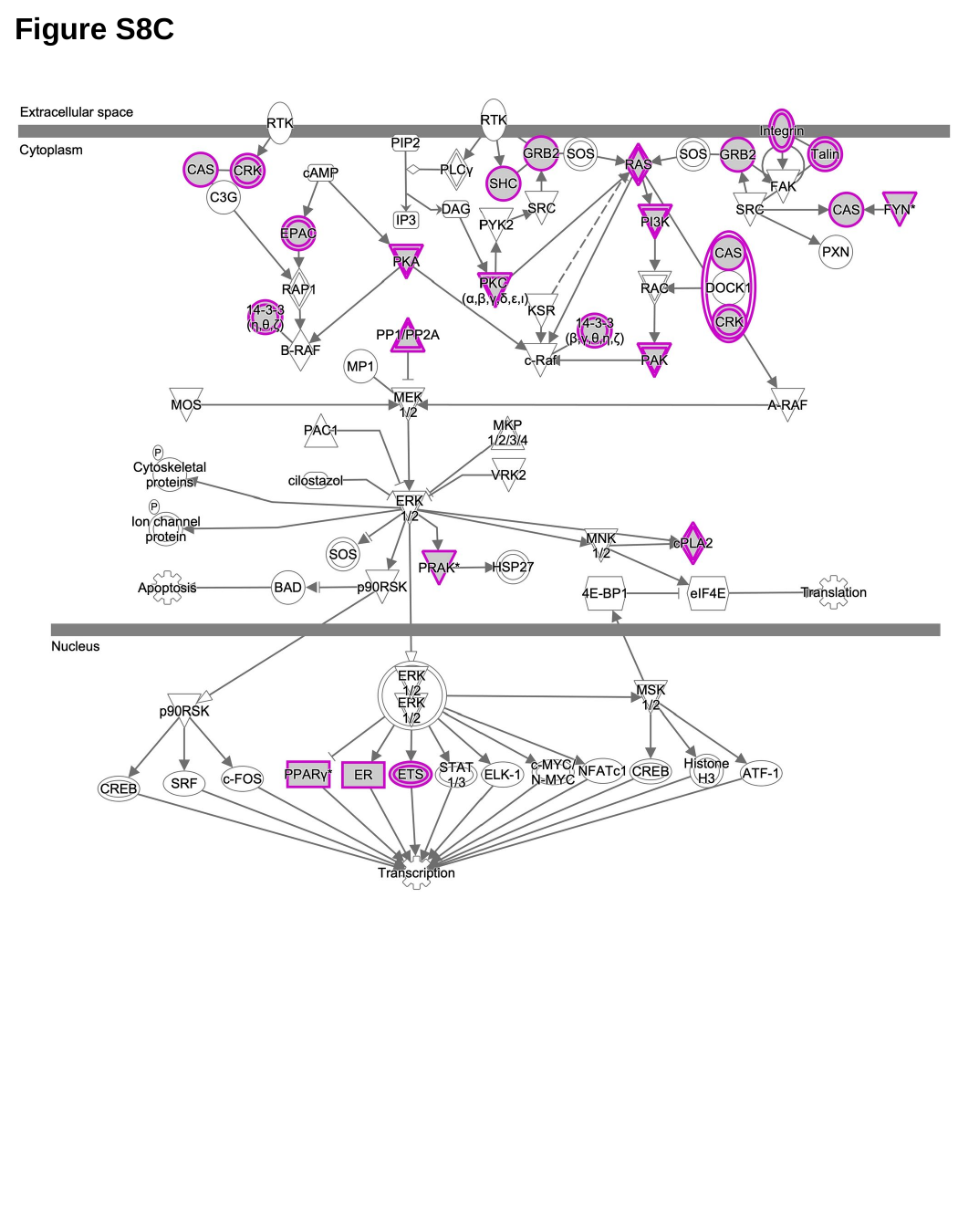

Figure S8C

## Slide 18
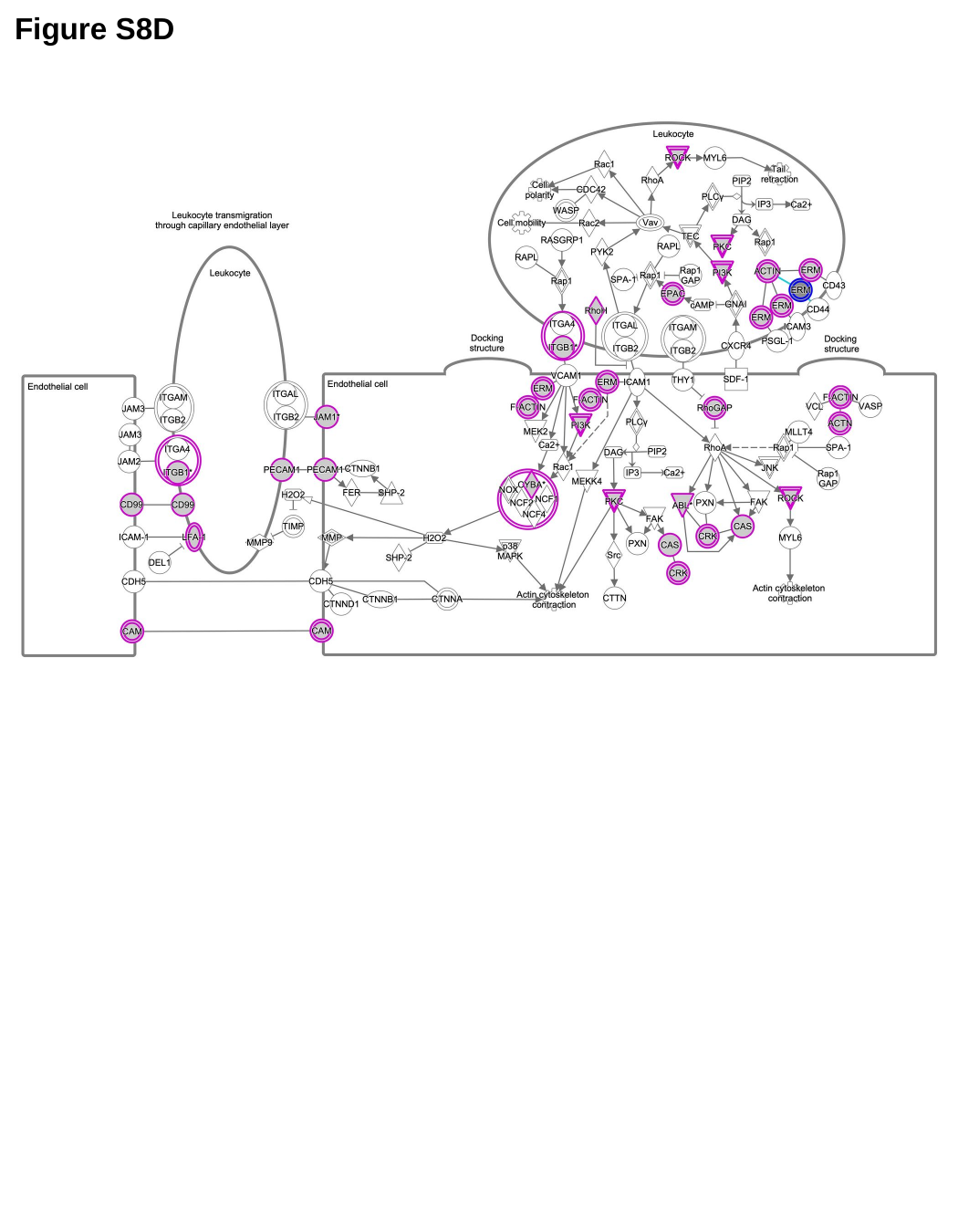

Figure S8D

## Slide 19
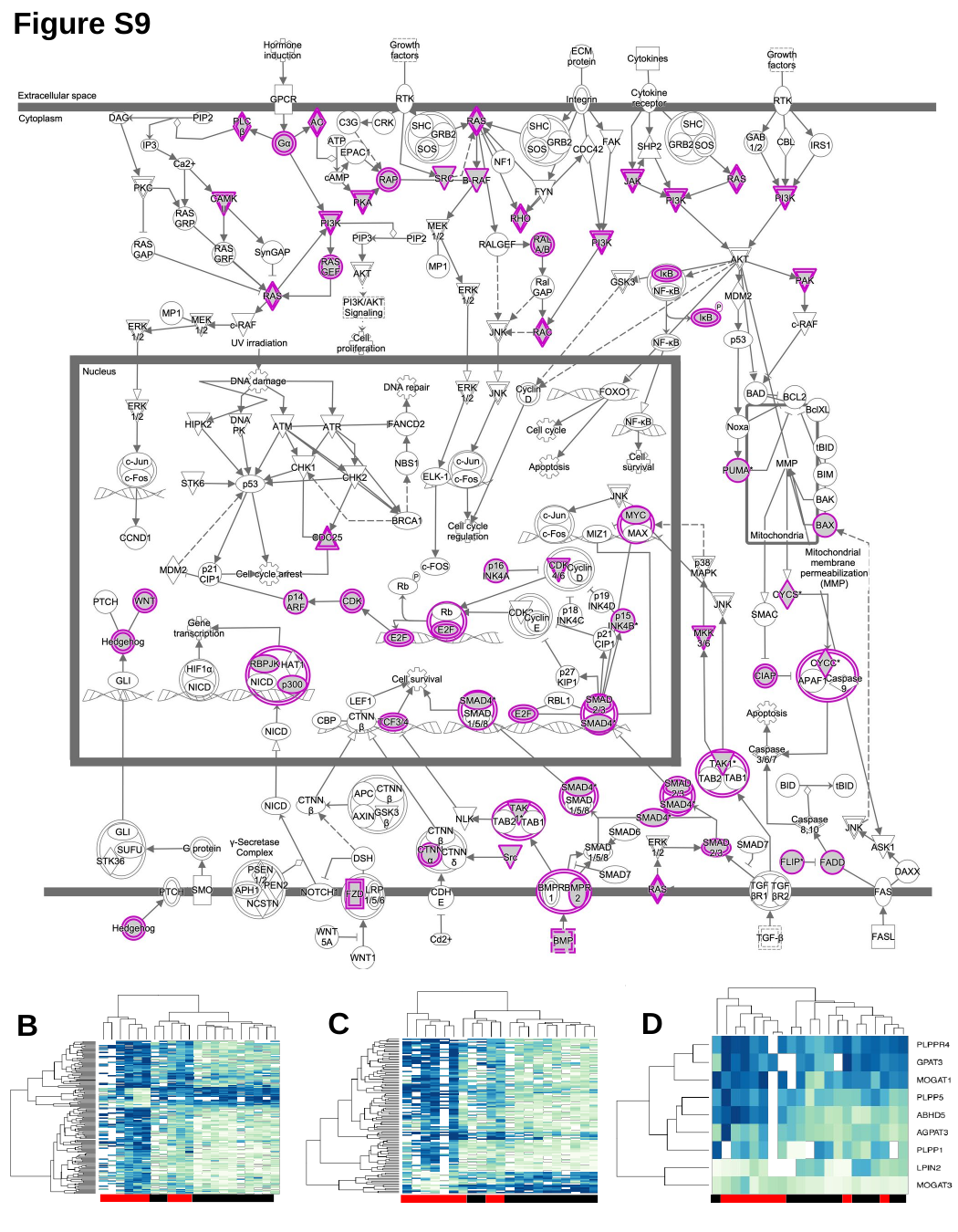

Figure S9
C
D
B

## Slide 20
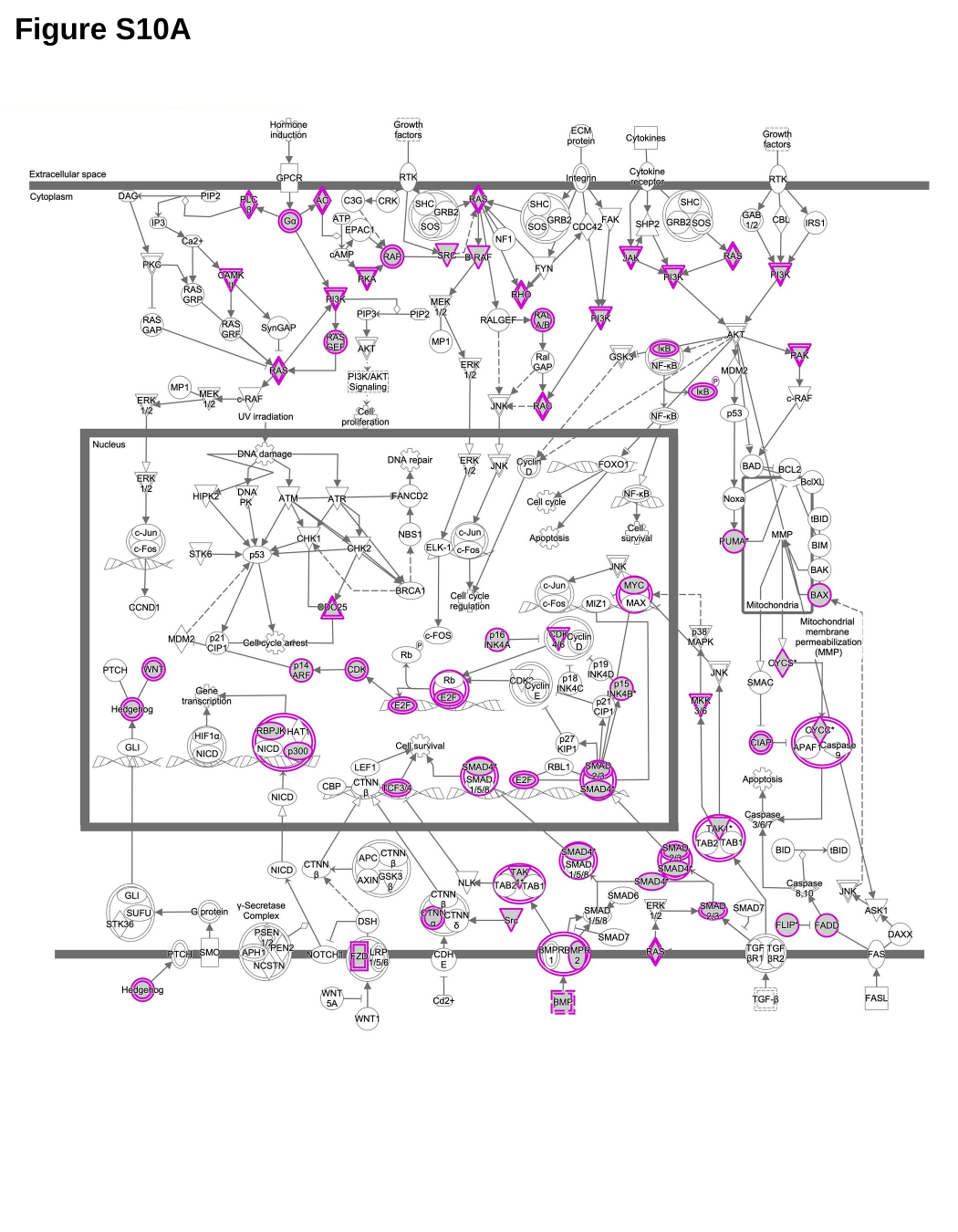

Figure S10A

## Slide 21
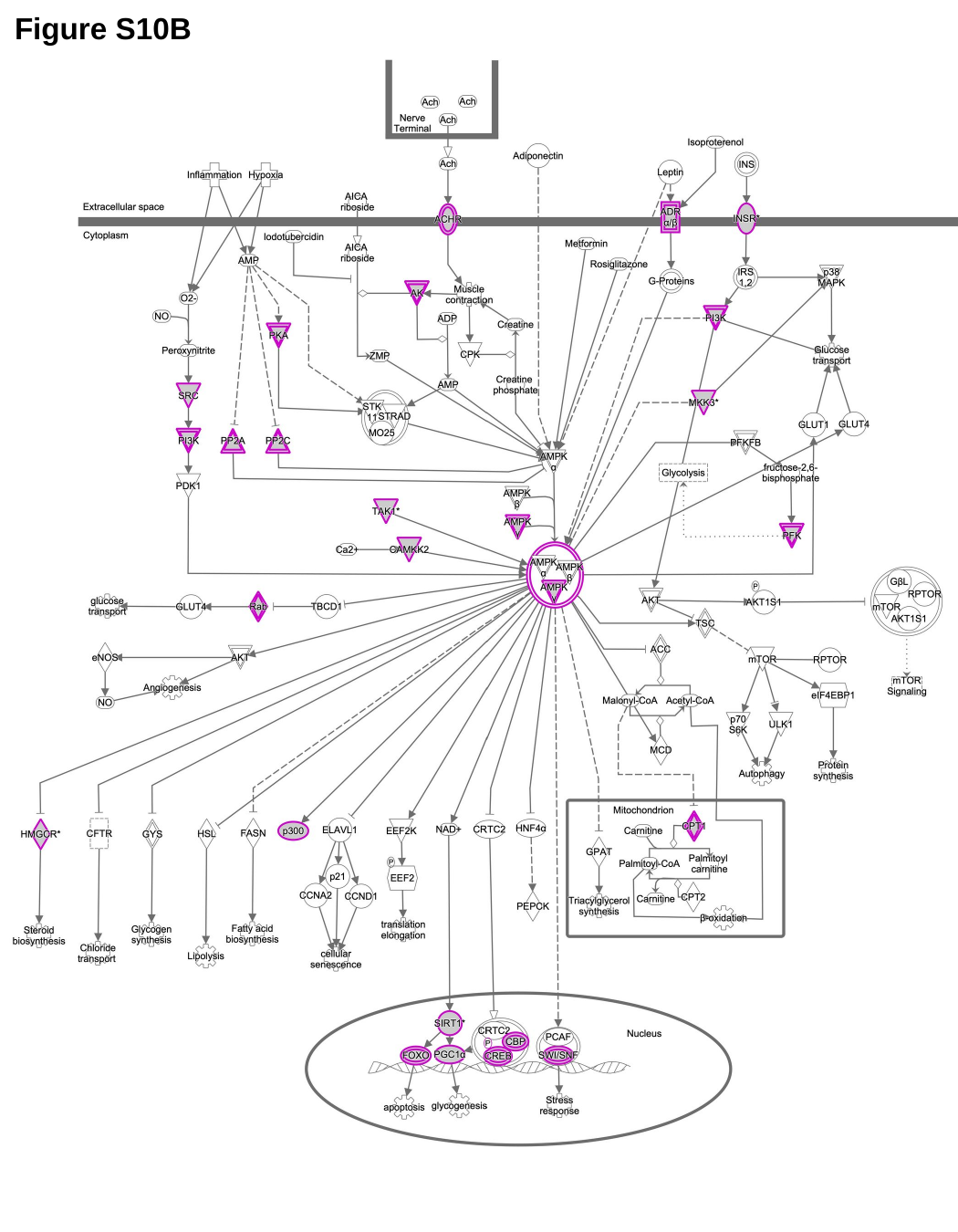

Figure S10B

## Slide 22
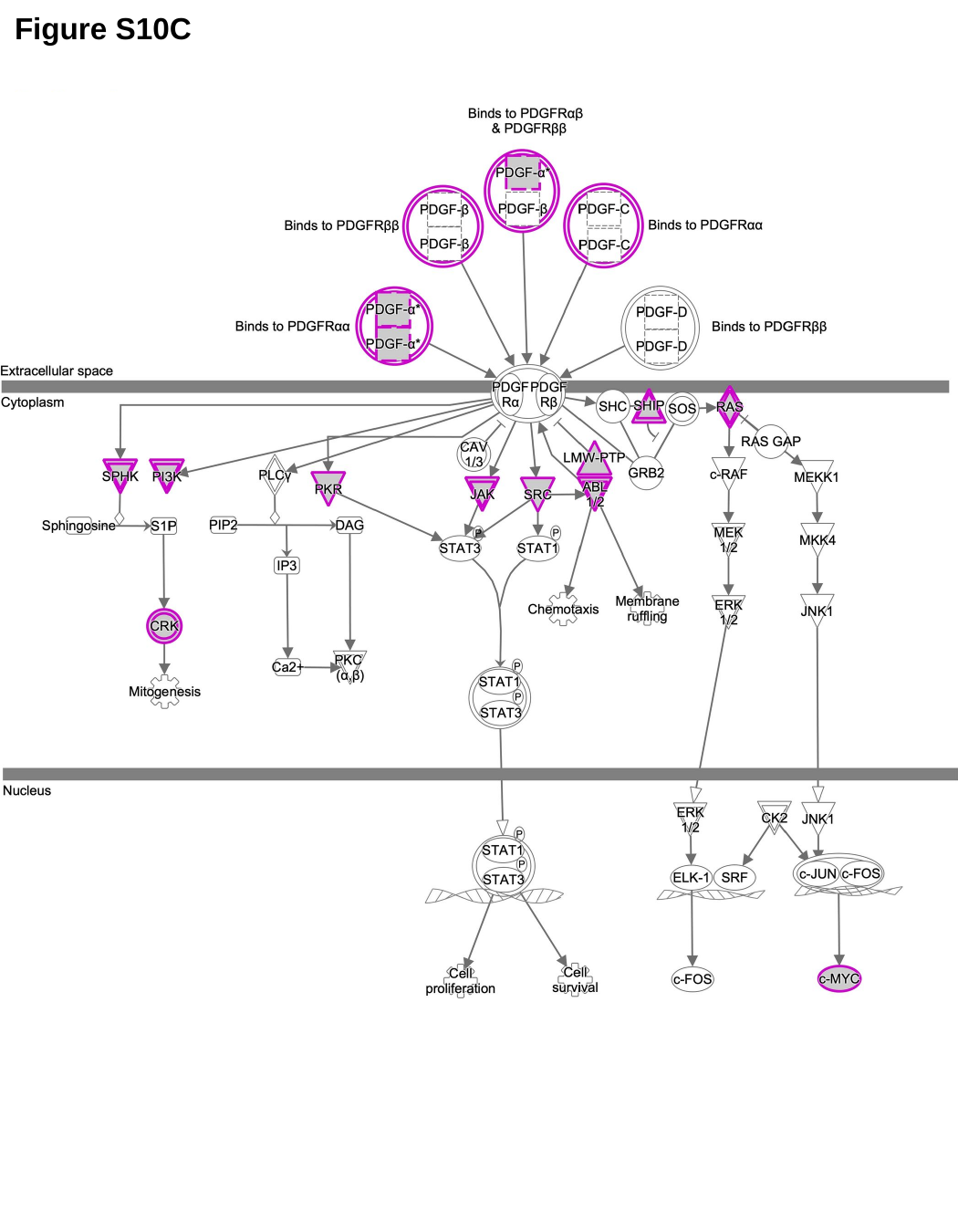

Figure S10C

## Slide 23
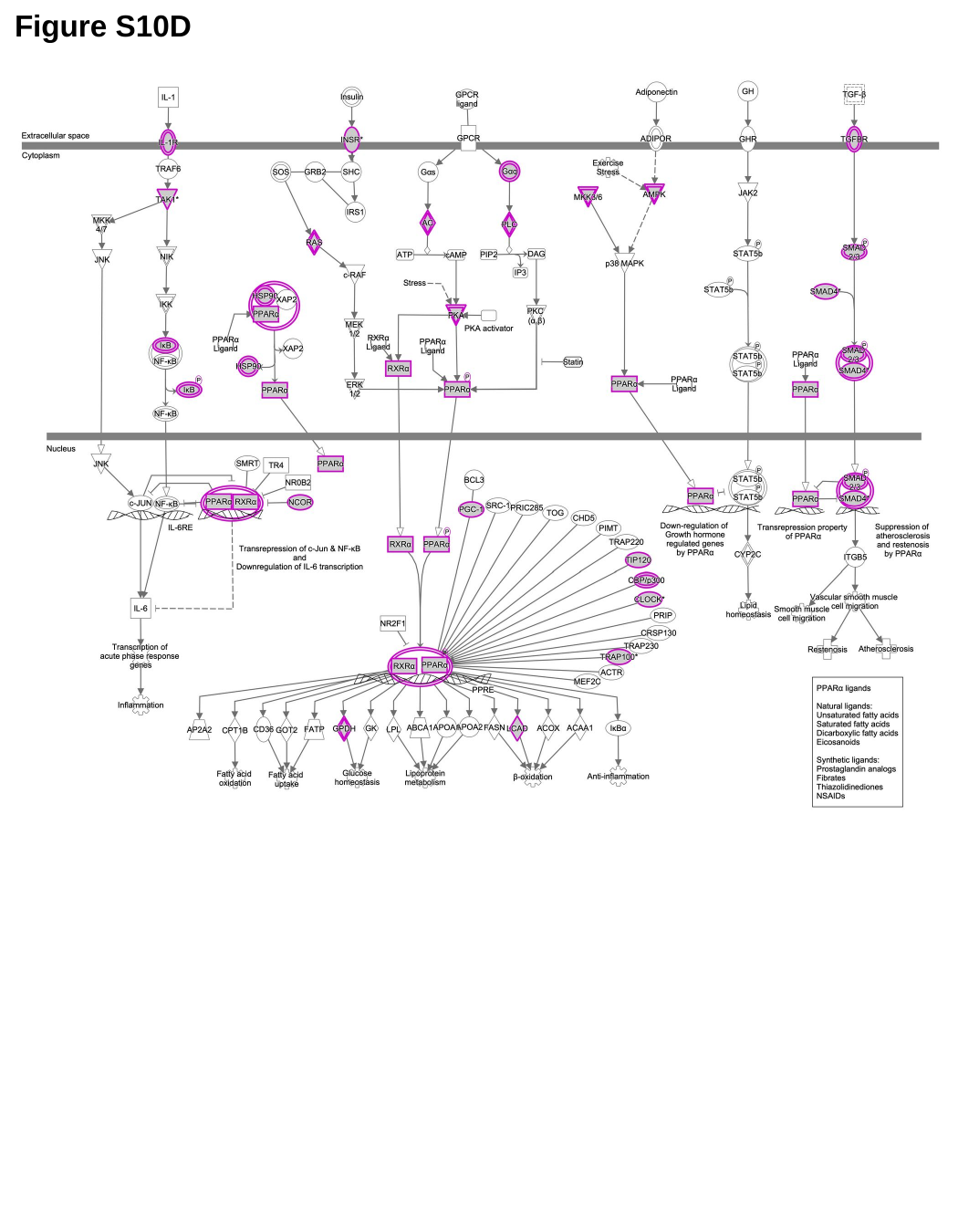

Figure S10D

## Slide 24
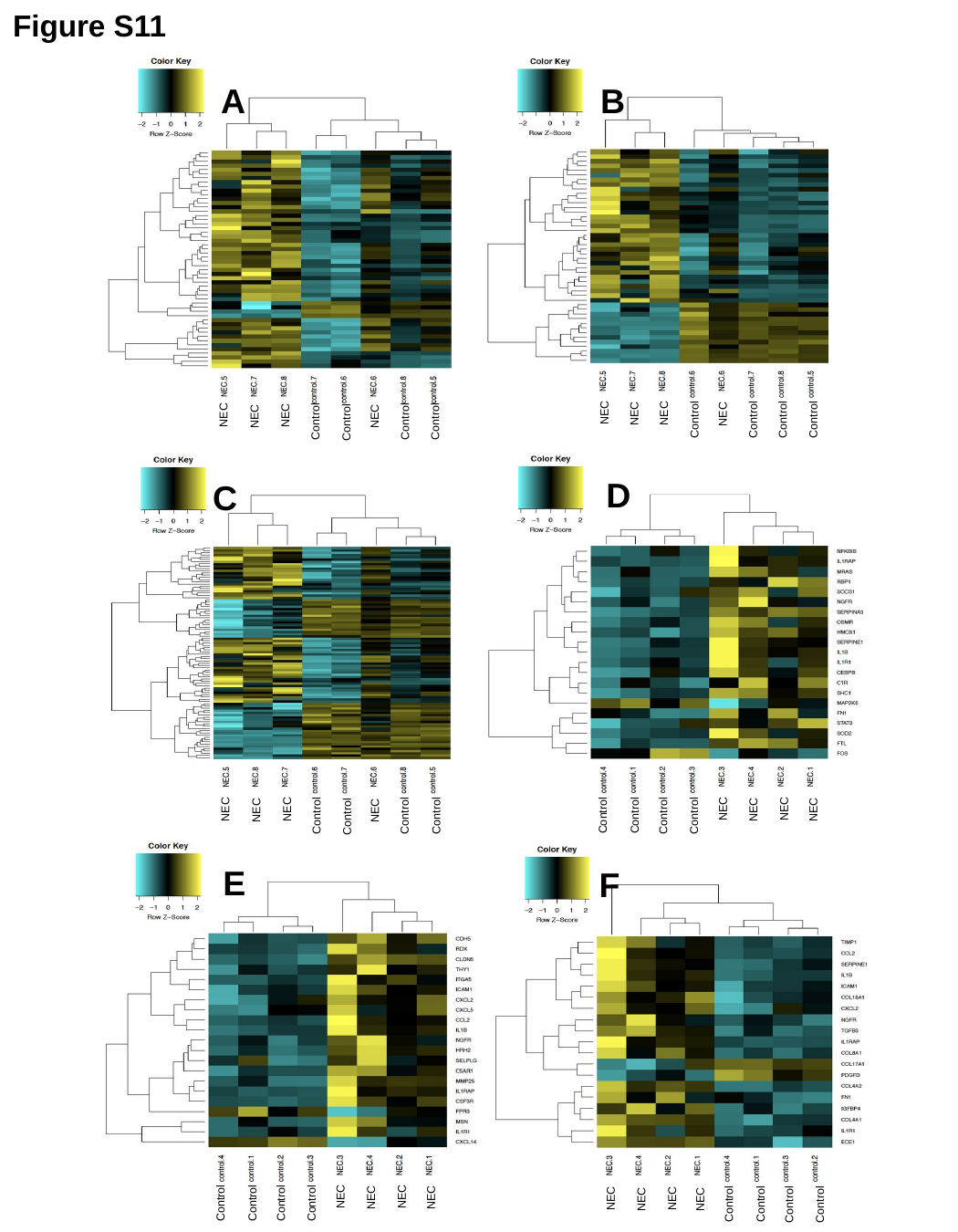

Figure S11
A
B
NEC
Control
Control
Control
NEC
Control
NEC
NEC
NEC
Control
Control
Control
NEC
NEC
Control
NEC
C
D
NEC
Control
Control
NEC
NEC
Control
Control
Control
Control
Control
NEC
NEC
NEC
NEC
Control
NEC
E
F
NEC
Control
Control
NEC
Control
Control
NEC
NEC
Control
Control
Control
Control
NEC
NEC
NEC
NEC

## Slide 25
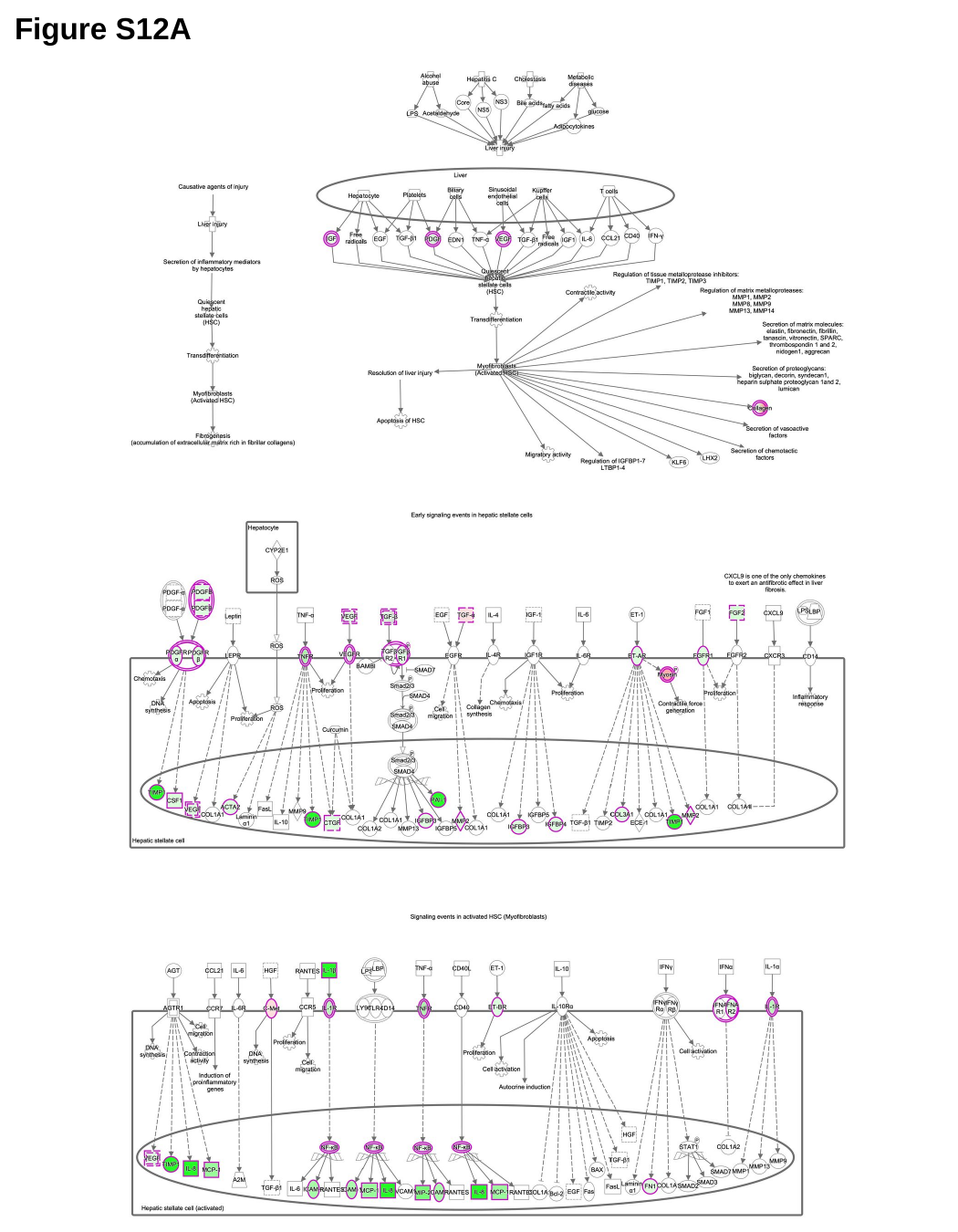

Figure S12A

## Slide 26
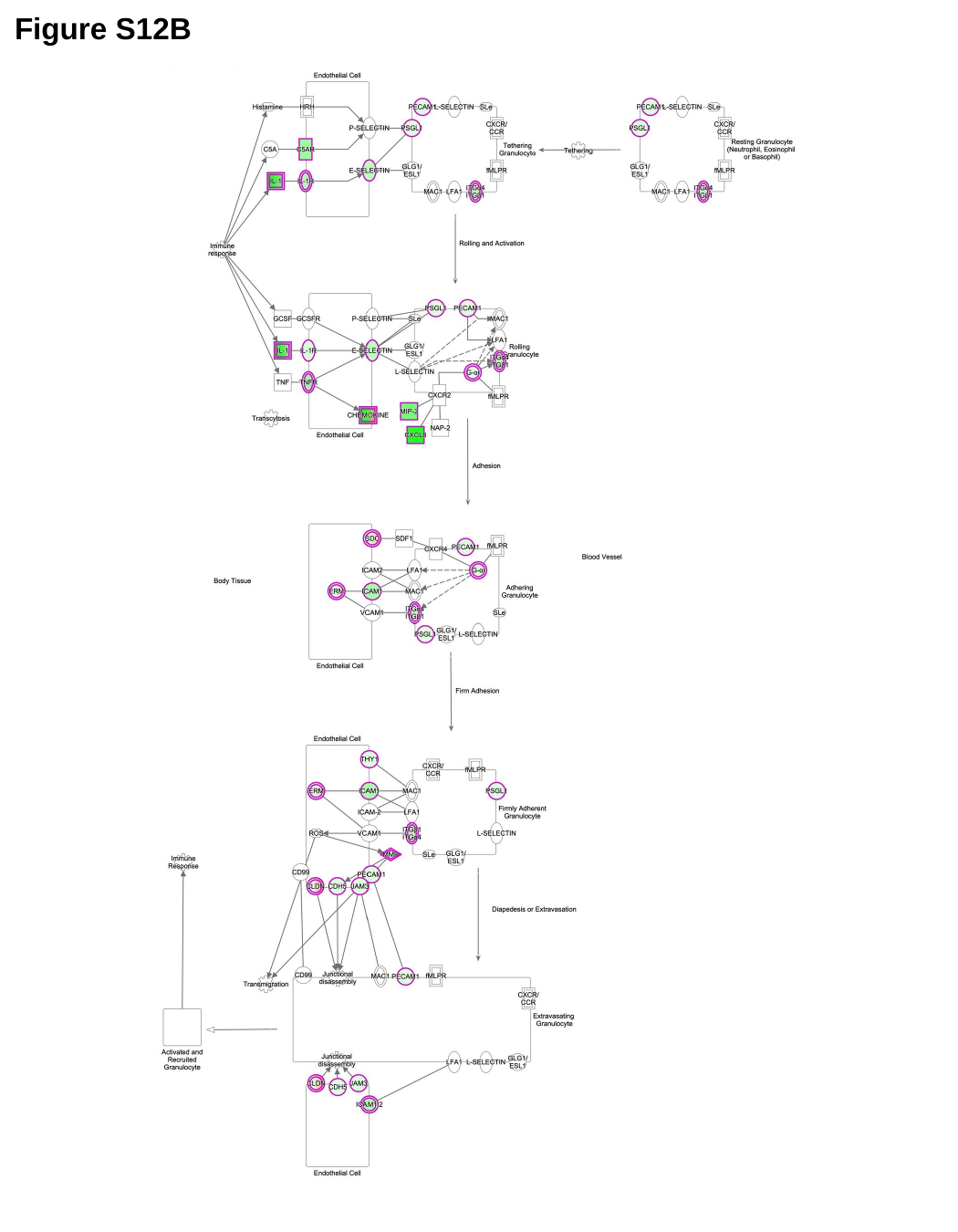

Figure S12B

## Slide 27
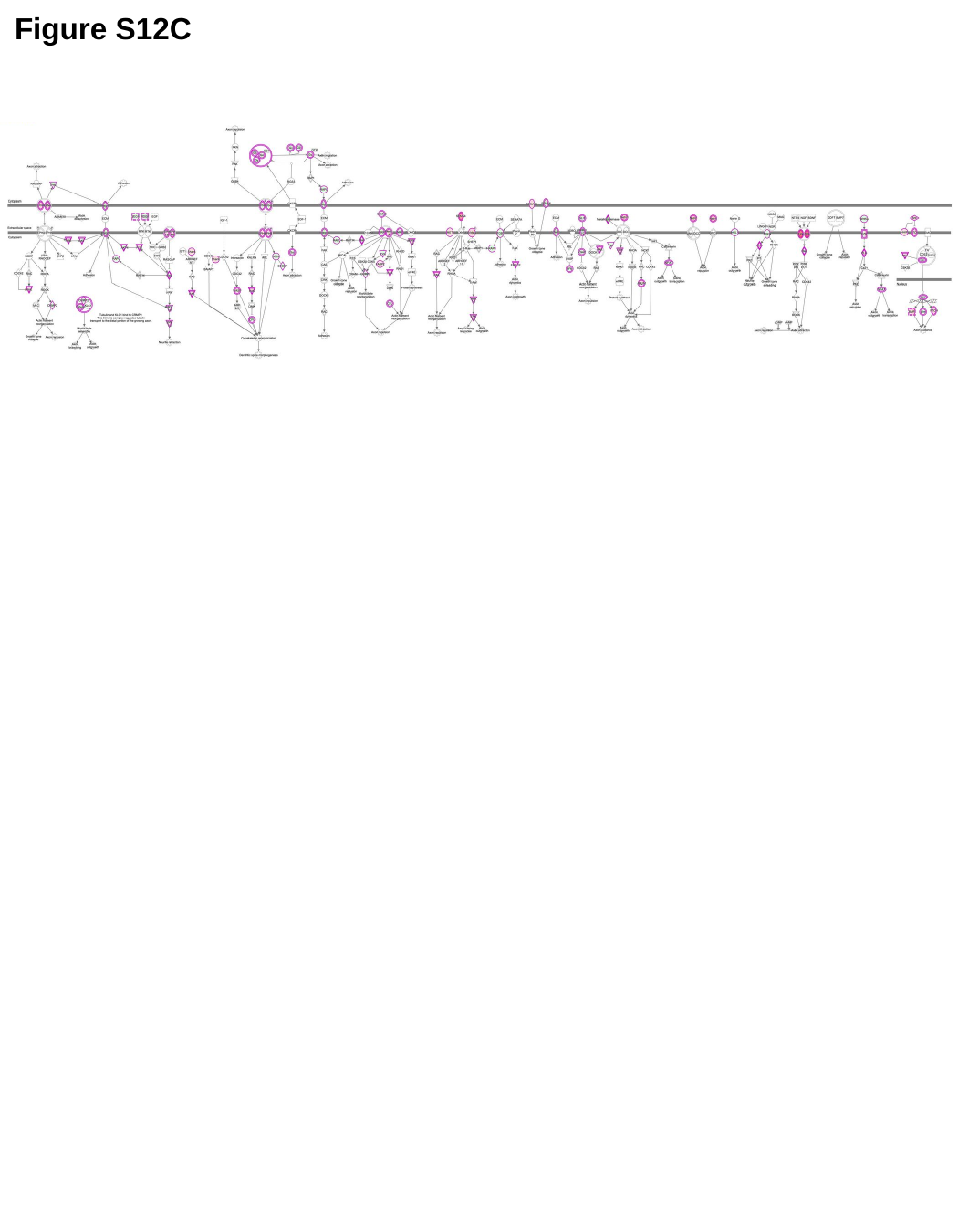

Figure S12C

## Slide 28
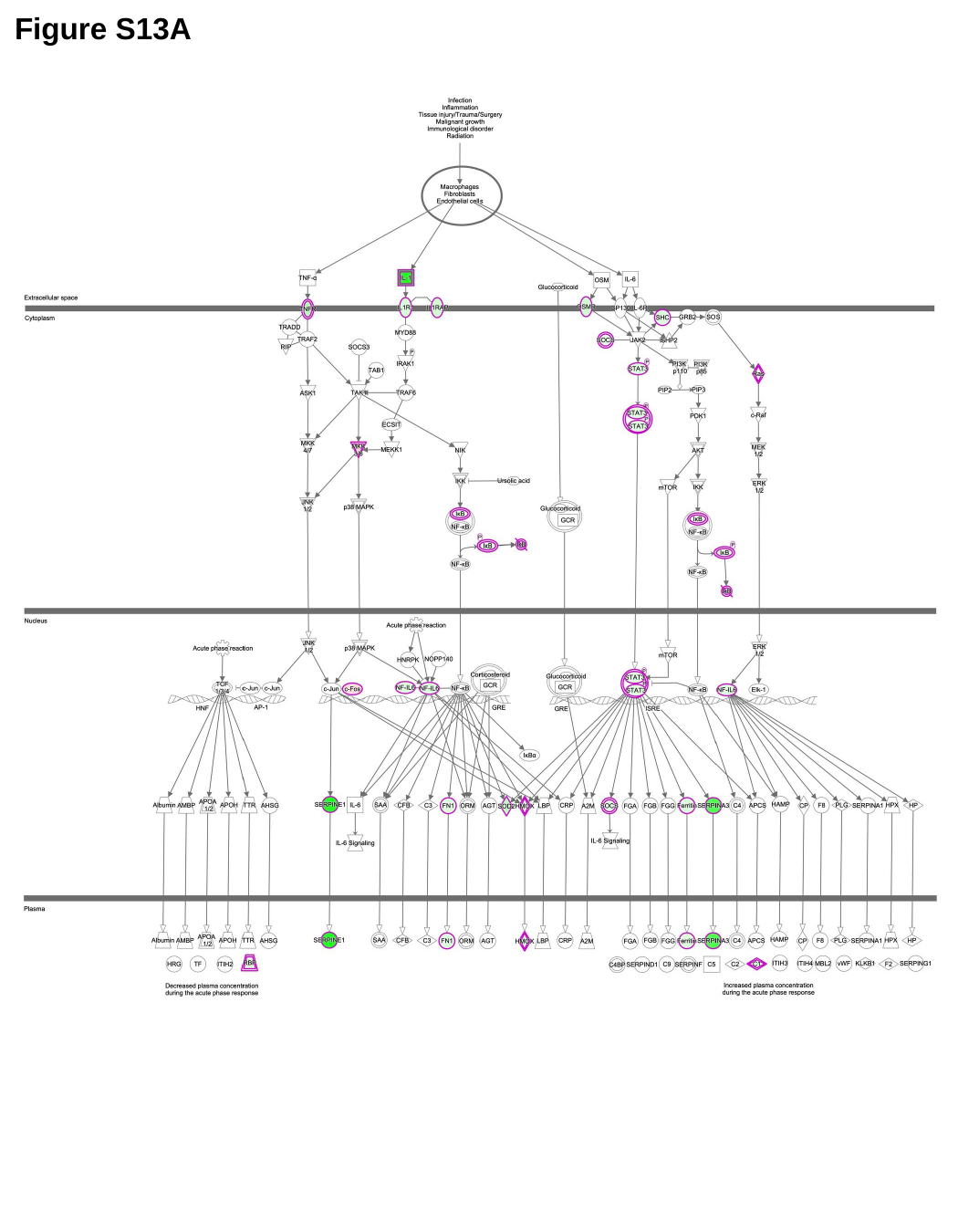

Figure S13A

## Slide 29
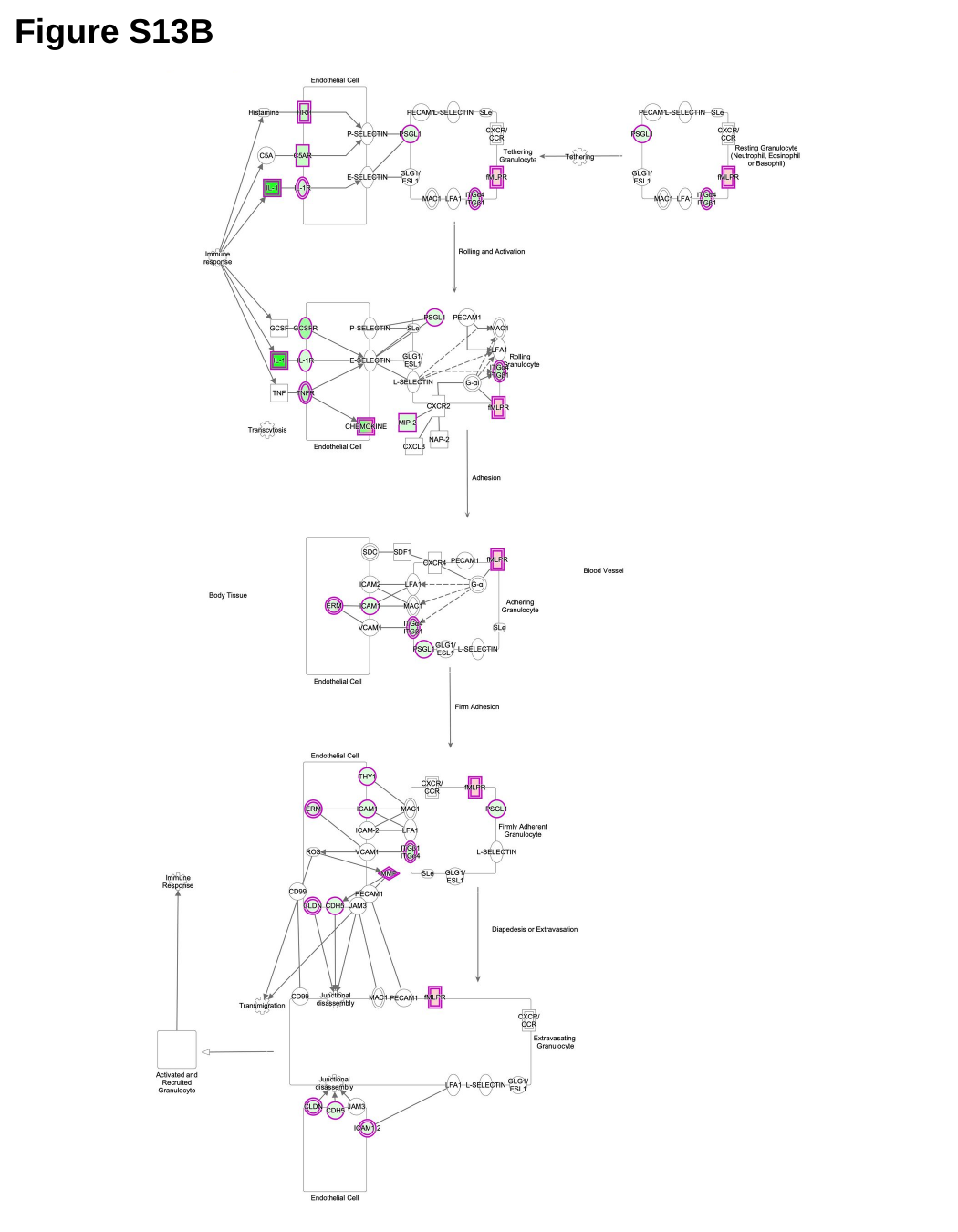

Figure S13B

## Slide 30
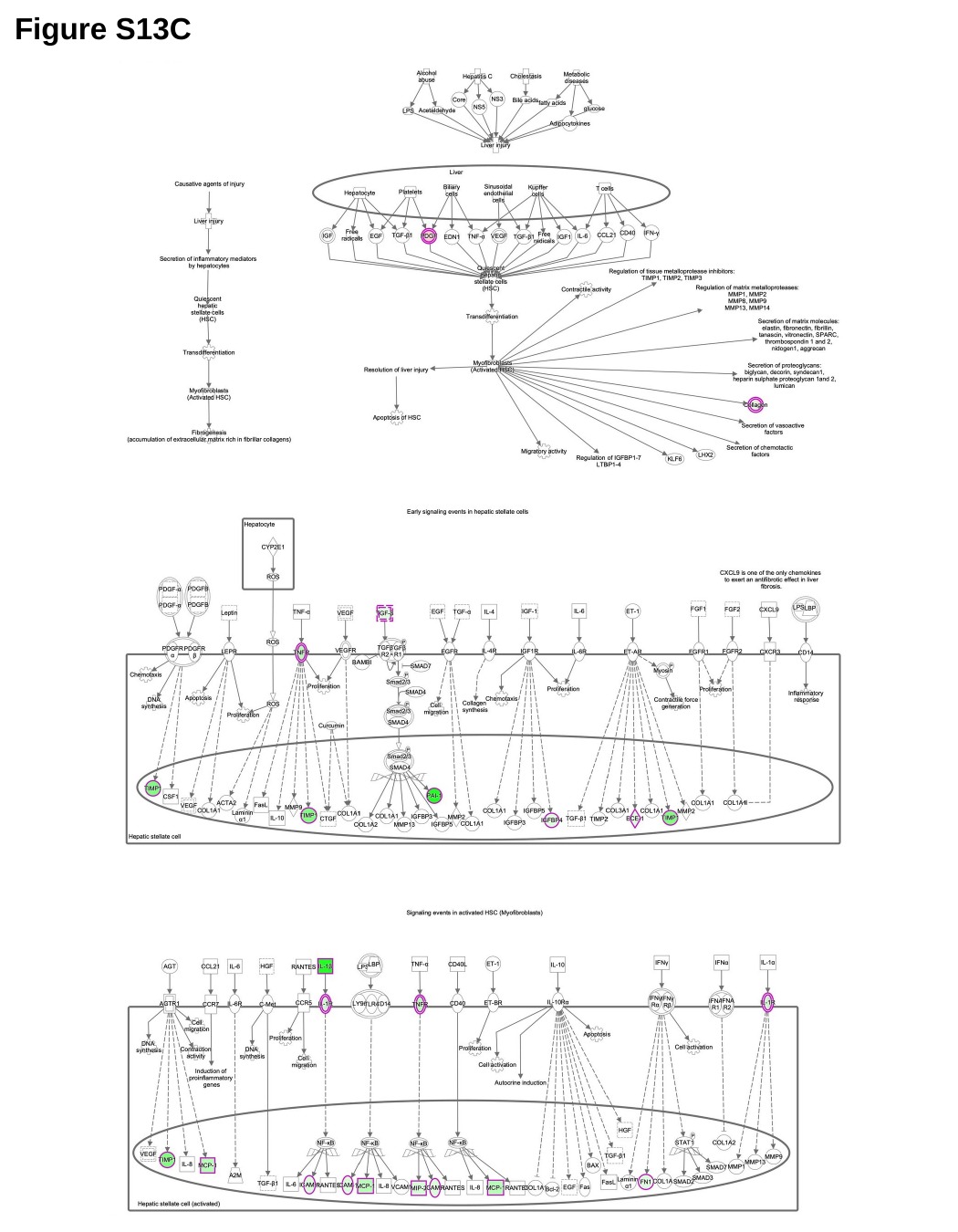

Figure S13C

## Slide 31
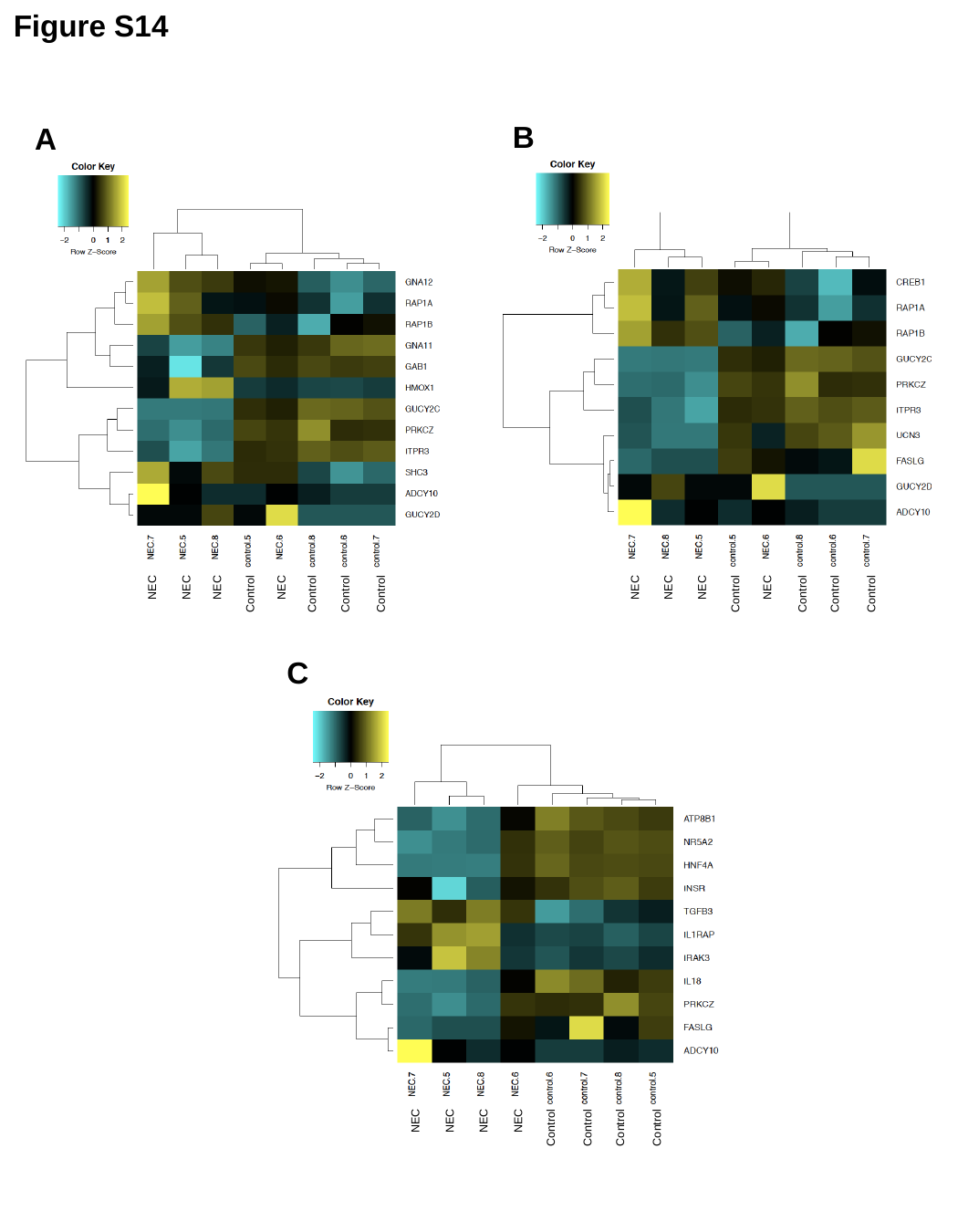

Figure S14
B
A
NEC
NEC
NEC
Control
NEC
Control
Control
Control
NEC
NEC
NEC
Control
NEC
Control
Control
Control
C
NEC
NEC
NEC
NEC
Control
Control
Control
Control
